# Have GRACE satellites overestimated groundwater depletion in the Northwest India Aquifer?

**DOI:** 10.1038/srep24398

**Published:** 2016-04-14

**Authors:** Di Long, Xi Chen, Bridget R. Scanlon, Yoshihide Wada, Yang Hong, Vijay P. Singh, Yaning Chen, Cunguang Wang, Zhongying Han, Wenting Yang

**Affiliations:** 1 State Key Laboratory of Hydroscience and Engineering, Department of Hydraulic Engineering, Tsinghua University, Beijing 100084, China; 2 Bureau of Economic Geology, Jackson School of Geosciences, The University of Texas at Austin, Austin, TX 78758, United States; 3 Department of Physical Geography, Utrecht University, Utrecht, Netherlands; 4 NASA Goddard Institute for Space Studies, New York, NY 10025, United States; 5 Center for Climate Systems Research, Columbia University, New York, NY, United States; 6 International Institute for Applied Systems Analysis, Laxenburg, Austria; 7 Department of Civil Engineering and Environmental Science, University of Oklahoma, Norman, OK 73072, United States; 8 Department of Biological & Agricultural Engineering, Texas A&M University, College Station, TX 77843, United States; 9 Department of Civil & Environmental Engineering, Texas A&M University, College Station, TX 77843, United States; 10 State Key Laboratory of Desert and Oasis Ecology, Xinjiang Institute of Ecology and Geography, Chinese Academy of Sciences, Urumqi, Xinjiang 830011, China

## Abstract

The Northwest India Aquifer (NWIA) has been shown to have the highest groundwater depletion (GWD) rate globally, threatening crop production and sustainability of groundwater resources. Gravity Recovery and Climate Experiment (GRACE) satellites have been emerging as a powerful tool to evaluate GWD with ancillary data. Accurate GWD estimation is, however, challenging because of uncertainties in GRACE data processing. We evaluated GWD rates over the NWIA using a variety of approaches, including newly developed constrained forward modeling resulting in a GWD rate of 3.1 ± 0.1 cm/a (or 14 ± 0.4 km^3^/a) for Jan 2005–Dec 2010, consistent with the GWD rate (2.8 cm/a or 12.3 km^3^/a) from groundwater-level monitoring data. Published studies (e.g., 4 ± 1 cm/a or 18 ± 4.4 km^3^/a) may overestimate GWD over this region. This study highlights uncertainties in GWD estimates and the importance of incorporating a priori information to refine spatial patterns of GRACE signals that could be more useful in groundwater resource management and need to be paid more attention in future studies.

Groundwater is a valuable resource for sustaining agricultural, industrial, and domestic water use in many arid and semi-arid regions globally^1^. Irrigation consumed ~90% of global freshwater resources during the past century, with ~40% of irrigation derived from groundwater, and groundwater use in irrigation has markedly increased^2^. Aquifer overexploitation within the context of population growth and climate extremes (e.g., droughts) has resulted in significant groundwater depletion (GWD), e.g., ~330 km^3^ over the High Plains (HP) Aquifer in the US from predevelopment (1950s) through 2013 representing ~8% of groundwater storage available before irrigation, and ~140 km^3^ over the California Central Valley (CV) from models since the 1860s through 2003^3^. GWD is threatening sustainable groundwater use and may compromise future agricultural production. Negative environmental impacts of GWD include decreased baseflow that may lead to drying-up of wetlands and rivers, land subsidence, saltwater intrusion, increasing pumping costs, and declining water supplies for some of the world agricultural areas putting sustained crop production at risk^4^. Accurate monitoring of both the rate and spatial pattern of GWD is imperative to formulate reasonable policies to achieve the goals of maintaining agricultural production and sustainable groundwater resources management^5^,^6^.

Traditional approaches for assessing GWD rely on groundwater-level data from wells that are relatively sparse or not accessible in most aquifers globally, limiting holistic and consistent assessments of GWD over major aquifers that are extremely important for agricultural production^7^. Global hydrological models (GHMs) are powerful tools for evaluating GWD over aquifers, e.g., the WaterGAP Global Hydrological Model (WGHM)^8^,^9^ and PCR-GLOBWB^10^,^11^,^12^. Since its launch in 2002, Gravity Recovery and Climate Experiment (GRACE) satellites provide an unprecedented opportunity to capture the variability in the Earth’s gravity field that is induced primarily by changes in total water storage (TWS) integrating changes in surface water storage (SWS) (e.g., canopy, reservoirs, wetlands and lakes, rivers, and snow water equivalent), soil moisture storage (SMS), and groundwater storage (GWS)^13^, making monitoring GWD in major aquifers globally possible. GHMs and GRACE satellites are complementary, joint use of which may improve estimates of TWS changes and GWD^14^.

GWS changes are often disaggregated from GRACE TWS changes by subtracting monitored SWS changes (e.g., changes in reservoir storage) and simulated SMS changes (e.g., land surface models, LSMs). In some cases, changes in SWS may be negligible accounting for a relatively small proportion of TWS changes^15^,^16^. The spatial resolution of GRACE signals is relatively low (~200,000 km^2^), limited by its altitude of ~450 km × 450 km^17^; however, large mass changes in aquifers smaller than the GRACE footprint (e.g., 52,000 km^2^ for the CV) due to irrigation pumpage allow storage changes to be detected by GRACE^18^. There have been great efforts made to estimate GWD over aquifers globally using GRACE in combination with ancillary data, e.g., the HP Aquifer^3^,^15^ and California’s CV^7^,^18^ in the US, the Middle East^19^,^20^, the Northwest India Aquifer (NWIA)^16^,^21^, and the North China Plain^22^,^23^. Recent studies provide holistic assessments of groundwater stress and resilience over aquifers globally; however, uncertainties in groundwater storage should be evaluated in detail to assess the remaining lifespan of some critical depleting aquifers^24^,^25^.

The NWIA has been shown to experience among the highest GWD rates globally over the past decade based on GHM simulations or GRACE observations^26^. One of the most important studies on GWD over the NWIA was conducted by *Rodell et al*.^16^ that indicated a GWD rate of 4 ± 1 cm/a for the three-state region in Northwest India during Aug 2002–Oct 2008 from GRACE spherical harmonic (SH) solutions (Release No.4, RL04) in combination with SMS changes from LSMs in Global Land Data Assimilation System-1 (GLDAS)^27^. Human-induced variability (28% of the area was irrigated and 95% of water consumption was attributed to irrigation) instead of natural variability dominated GWD in this region. However, as *Rodell et al*.^16^ indicated, the GRACE-derived GWD rate was higher than the difference between annual available recharge and annual withdrawals in the three-state region of 13.2 km^3^/a (equivalent to 3 cm/a) reported by the Indian Ministry of Water Resources. The lower GWD rate of 3 cm/a was presumably attributed to underestimation of withdrawals and/or overestimation of recharge from irrigated water by the Indian government^16^. Other GRACE-based studies provided GWD rates ranging from 2.0 ± 0.3 cm/a over an ~2,700,000 km^2^ region of North India, Afghanistan, Pakistan, Nepal and Bangladesh from Apr 2003 through Jun 2008^28^, to 2.1 ± 0.7 cm/a from Jan 2003 through Dec 2012^21^, 1.9 cm/a for the period Jan 2003–Dec 2010^29^, and even only 1.5 cm/a for Jan 2003 to Dec 2012^30^ over different sub-regions in North India. Disparities in these GWD estimates reflect differences in study boundary, time period, and more importantly GRACE data processing algorithms. This makes it imperative to compare GRACE-based GWD with ground-based data to assess their validity; however, published studies on GRACE-based GWD estimates in the NWIA have not been compared with detailed ground-based monitoring data.

GRACE satellites are relatively new and data processing is continually evolving. Processing GRACE data generally requires noise reduction at high frequencies using low-pass filtering (e.g., truncation of SH at the maximum degree and order of 60, destriping, and 300 km Gaussian filtering), which inevitably leads to signal loss^31^ that needs to be restored using either synthetic data commonly obtained from LSMs, or other techniques depending less on LSM output. However, the degree of signal restoration is extremely critical, varying with GRACE SH solutions, processing approaches and their assumptions, and the distribution of water storage changes for a specific region as well. The latest version of the SH solution of the Earth’s gravity field from GRACE (Center for Space Research, CSR RL05) further reduces uncertainties in TWS change by up to 40%^32^. Examining GWD rates using the latest SH solution and different approaches for the same region and period in the NWIA and evaluating GWD estimates with groundwater-based monitoring data are imperative to develop a thorough understanding of uncertainties in restored GWD rates from different approaches and how much groundwater has really been depleted in the NWIA.

Typical approaches for restoring GRACE signals can be categorized as: (1) approaches using water storage changes from LSMs, e.g., the scaling factor approach^33^ and additive approach^17^,^34^, and (2) approaches depending less on water storage changes from LSMs, e.g., the multiplicative approach^35^,^36^, and unconstrained or constrained forward modeling approaches^21^,^37^ (details about these approaches can be found in the Methods section and Supporting Information 1). Scaling factor, additive, and multiplicative approaches have been widely applied to restore GRACE signals for land hydrology. Unconstrained and constrained forward modeling has been shown to be effective in reducing the leakage effect over the cryosphere^37^. Unconstrained forward modeling, as used in *Chen et al*.^21^ to estimate GWD in the NWIA, includes iterative correction without using a priori knowledge about the spatial distribution of water storage variations. Therefore, it cannot recover the detailed spatial pattern of GWD and the leakage effect cannot be completely reduced. Constrained forward modeling uses a priori information regarding the spatial distribution of water storage changes to constrain the recovered signals. However, the utility of constrained forward modeling to resolve GWD in aquifers has not been examined.

We hypothesize that (1) the spatial distribution of water storage changes for a study region may impact the utility of both unconstrained and constrained forward modeling; and (2) integration of the spatial pattern of water storage changes, instead of the absolute estimates of GWD from synthetic data (e.g., GHM output) for restoring GRACE signals, may significantly improve delineation of the exact spatial pattern of GWD from GRACE satellites. We will: (1) test the impact of the distribution of groundwater storage changes on unconstrained and constrained forward modeling to recover signal loss due to low-pass filtering using synthetic data; (2) integrate the spatial pattern of GWD from a GHM, PCR-GLOBWB, to estimate GWD from GRACE and ancillary data for the three-state region of the NWIA, and (3) compare GRACE-based approaches (i.e., scaling factor, additive, multiplicative, and forward modeling approaches) for estimating GWD for the three-state region and compare GRACE-derived GWD with ground-based monitoring data.

## Results

**Figure 1:**
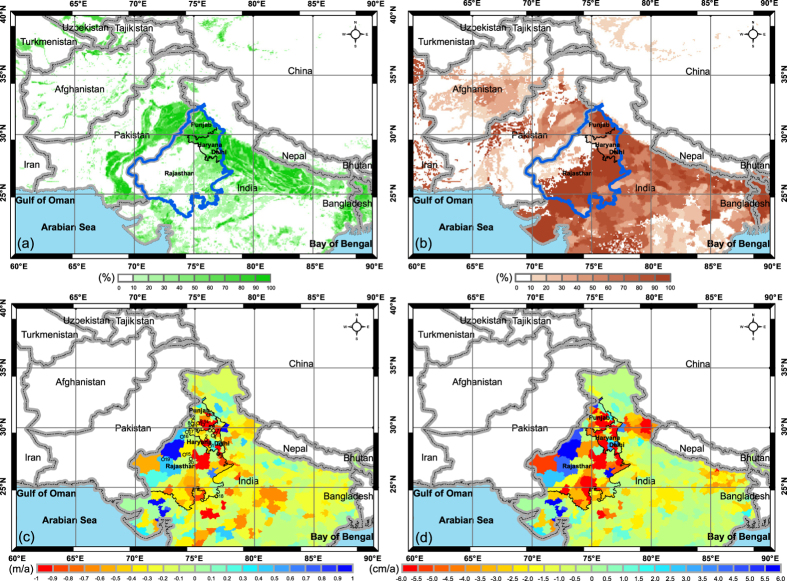
(**a**) Three-state region (Punjab, Haryana & Delhi, and Rajasthan) in India shown in blue polygon and areas equipped for irrigation (%) over North India, (**b**) areas equipped for irrigation with groundwater (%) from AQUASTAT data of the Food and Agricultural Organization (FAO) of the United Nations, (**c**) slopes of variations in groundwater level, and (**d**) GWD (cm/a) from groundwater-level monitoring data over the three-state region and its surroundings for the period 2005–2010. Open circles in Fig. 1(c) represent 20 selected groundwater monitoring sites in the three-state region, with groundwater-level time series for the 20 sites shown in Fig. S2. Map was created using ArcGIS (http://www.esri.com/software/arcgis/arcgis-for-desktop).

We use the three-state region (i.e., Punjab, Haryana & Delhi, and Rajasthan with a total area of 438,296 km^2^, the same as *Rodell et al*.^16^) in the NWIA as a test-bed, with ~30% of the study region equipped for irrigation (Fig. 1(a)) and 74% of the equipped area irrigated with groundwater (Fig. 1(b) and Supporting Information 2). Note that only 16% of Rajasthan state was irrigated. The GWD rate from groundwater-level data for the six-year period (Jan 2005–Dec 2010, Fig. 1c) and specific yield (Fig. S1) for the three-state region was estimated to be 2.8 cm/a (Fig. 1d and Supporting Information 2, GWD shown in negative in all figure legends). Variations in groundwater levels for most of 20 selected sites across the three states show rapidly declining trends (Fig. S2), particularly in the east but much less in the west, with even some recovery of groundwater levels in the west (Fig. 1c,d). Specific yield values to convert groundwater level changes to storage for unconfined aquifers for Punjab and Haryana were estimated to be ~0.15^38^, which is dominated by sandy alluvium (a specific yield of 0.15) that accounts for ~98% over the two states^39^. The specific yield in Rajasthan was estimated to be ~0.1, which is dominated by sandy alluvium (60%) and sandstone (11%) with a lower specific yield of 0.045 (Supporting Information 2).

### Evaluation of forward modeling using hypothetical data

A range of numerical simulations using hypothetical data was performed to evaluate the utility of unconstrained and constrained forward modeling to recover signal loss due to low-pass filtering. Constrained forward modeling can completely recover the signal loss during low-pass filtering, regardless of signal configuration, whereas unconstrained forward modeling cannot (Table S1). Expanding the study region to include the surrounding areas may allow one to obtain the total volume of GWD from the simulated GWD rates irrespective of signal configuration. In terms of spatial pattern, unconstrained forward modeling is unable to recover detailed spatial patterns of GWD, showing correlation coefficients between simulated and original GWD rates ranging from 0.04 for the completely random distribution to 0.22 for the uniform distribution (Table S1, Figs S3–S7). However, constrained forward modeling can recover the spatial pattern of GWD to varying degrees, showing correlation coefficients between the simulated and original GWD rates ranging from 0.36 for the completely random distribution to 0.99 for the uniform distribution. For the other three signal configurations, the correlation coefficients are slightly less than 0.5 (Table S1 and Figs S8–S12).

We conclude that the capability of constrained forward modeling to recover the detailed spatial distribution of signal depends highly on the spatial pattern of original data. The greater the heterogeneity in the signal, the lower the spatial correspondence between the simulated and original signals. The study by *Chen et al*.^37^ examined constrained forward modeling only with uniform distribution for mass change rates, i.e., a uniform mass change rate of −33 cm/a and −31 mm/a assigned to the Amundsen Sea Embayment and the Northern Peninsula, respectively, over the Antarctic region. While this approach may be suitable for this case because of relatively uniform distribution of glacier and ice melting over the Antarctic in cryosphere studies^37^,^40^, constrained forward modeling could not completely recover the spatial pattern of signals in land hydrology due to marked spatial heterogeneity in water storage changes over land. Results here using hypothetical data demonstrate that it is challenging to completely recover the spatial pattern of GWD rates if GWD is spatially heterogeneous.

### Evaluation of forward modeling using synthetic data from PCR-GLOBWB

**Figure 2:**
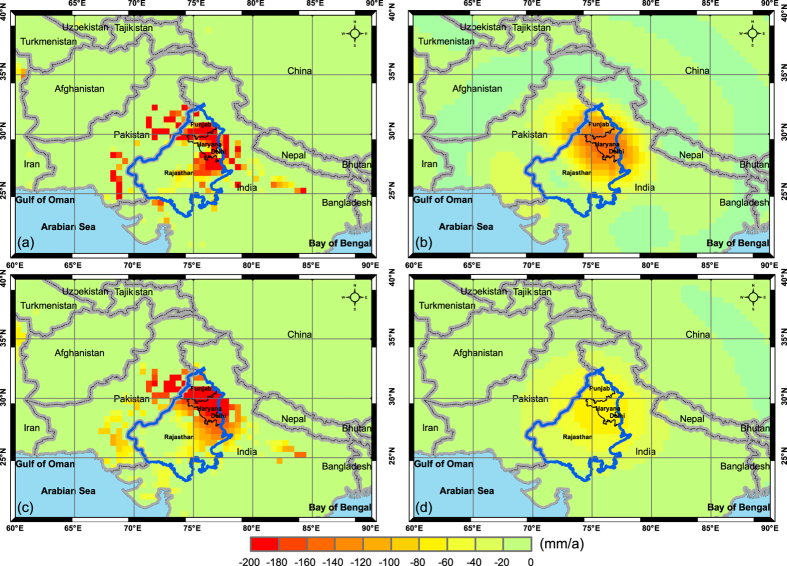
(**a**) Synthetical distributed GWD rates from PCR-GLOBWB for the period 2003–2010, (**b**) forward modeled GWD_s_ rate distribution after 500 iterations using unconstrained forward modeling, (**c**) forward modeled GWD_s_ rate distribution after 500 iterations using constrained forward modeling, and (d) filtered GWD rates from (**a–c**) after low-pass filtering. Map was created using ArcGIS (http://www.esri.com/software/arcgis/arcgis-for-desktop).

Both unconstrained and constrained forward modeling can recover over 95% of signal variations in GWD from PCR-GLOBWB over the three-state region for the period 2003–2010 (Table S1). Use of constrained forward modeling to recover filtered signals in GWD was confirmed again by using synthetic data from PCR-GLOBWB. Unlike the inability of unconstrained forward modeling to completely recover filtered signals using hypothetical data, it is interesting to see that unconstrained forward modeling can be effective (96%) in restoring the signal loss for the three-state region. For instance, GWD from PCR-GLOBWB for the period 2003 through 2010 was mainly distributed over the north and east of the three-state region, with an annual depletion rate of 6.7 cm/a (Fig. 2a). By performing low-pass filtering as applied to GRACE spherical harmonics, e.g., truncation at the maximum degree and order of 60 and 300 km Gaussian filtering, we obtained filtered GWD rates (Fig. 2d), with a significantly dampened GWD magnitude of 4.8 cm/a. In practice, we normally only have filtered GWD rates, e.g., from GRACE satellites and ancillary data, with the aim of obtaining the recovered GWD rates using the scaling factor, additive, or multiplicative approaches. Through unconstrained forward modeling, we can recover most of the dampened GWD rates, denoted as GWD_s_ in Fig. 2b, with a spatial mean of 6.4 cm/a. Therefore, unconstrained forward modeling can recover over 96% of the original GWD rates. By performing low-pass filtering for GWD_s_ (Fig. 2b), we can obtain filtered GWD rates that are the same as the filtered GWD rates obtained directly from PCR-GLOBWB (Fig. 2a).

The ability of unconstrained forward modeling to recover signal loss using PCR-GLOBWB can be attributed to signal amplification from the surrounding region that essentially reduces signal dampening within the three-state region. Though unconstrained forward modeling is unable to completely recover signal loss based on numerical simulations using hypothetical data where all signal variations are assumed to be completely restricted to the study region, there are some cases in practice that unconstrained forward modeling can generally recover signal loss when the study region is impacted by signal variations from its surroundings. Apparently, unconstrained forward modeling cannot recover the spatial pattern of GWD relative to the original pattern from synthetic data (Fig. 2b compared with Fig. 2a), showing a correlation coefficient between the simulated and PCR-GLOBWB GWD of 0.64 across the study region. Frequency distributions show that the simulated GWD rates with unconstrained forward modeling have a more even distribution (Fig. S13b) compared to the frequency distribution of GWD rates from PCR-GLOBWB that shows a sharp increase in the interval 0 to −10 mm/a dominated by areas without GWD (Fig. S13a). In particular, grid cells without GWD (e.g., surrounding those with GWD in Fig. 2a) do show GWD in Fig. 2b. This artifact resulted from signal leakage from the area with GWD to its surroundings due to low-pass filtering. Overall, without a priori information on the distribution of GWD, it is impossible to obtain a ‘realistic’ GWD distribution through unconstrained forward modeling.

The distribution of PCR-GLOBWB GWD is highly consistent with the GWD distribution from another GHM, WGHM (Fig. S14), the distribution of areas equipped for irrigation (Fig. 1a,b) and land cover (Fig. S15). It is apparent that the southwest of the three-state region is dominated by natural vegetation that is not equipped for irrigation, and therefore should not exhibit marked GWD. Globally constrained forward modeling (referring to the Methods section) can recover both the magnitude and spatial pattern of GWD relative to the original GWD from PCR-GLOBWB, showing a spatially averaged GWD rate of 6.8 cm/a and a correlation coefficient of 0.77 (Table S1 and Fig. 2c). This means that given the filtered GWD (e.g., Fig. 2d) and its spatial pattern (e.g., GWD rate < 0 in Fig. 2a), it is possible to completely recover the magnitude and generally recover the spatial pattern of GWD from globally constrained forward modeling. However, if one does not have the critical information on the spatial pattern of GWD, use of constrained forward modeling to recover GWD in both magnitude and spatial pattern may fail. This is demonstrated by the case of locally constrained forward modeling (referring to the Methods section) that assumes GWD to be restricted to the three-state region, showing a resulting magnitude of GWD of 12.2 cm/a and a correlation coefficient of 0.28 (Table S1 and Fig. S16). The assumption of GWD restricted to the three-state region used in previous studies is unreasonable because of large GWD across the surrounding areas, i.e., Pakistan and west Uttar Pradesh (Figs 1d and 2a).

### Groundwater storage depletion from GRACE using forward modeling

**Figure 3:**
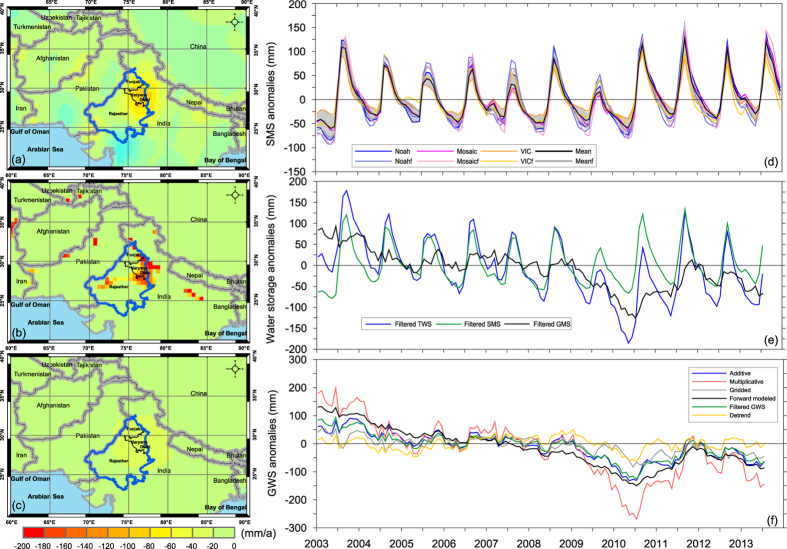
(**a**) GWD rates from original GRACE TWS changes without filtering minus GLDAS SMS changes for the period 2003–2010; (**b**) GWD_s_ rates from filtered GRACE GWD rates and globally constrained forward modeling after 500 iterations using the spatial pattern of GWD rates from PCR-GLOBWB for the same period, (**c**) filtered GRACE GWD rates, i.e., GWD_a_, (**d**) time series of filtered and original SMS changes from GLDAS-1 Noah, Mosaic, and VIC models, (**e**) filtered TWS changes from GRACE, filtered SMS changes from the mean of filtered SMS changes, and filtered GWS changes from filtered TWS changes minus filtered SMS, and (**f**) time series of restored GWS anomaly time series from the additive, multiplicative, gridded, and (globally constrained) forward modeling approaches. Also shown are the filtered GWS changes and filtered GWS changes whose trends were removed. Map was created using ArcGIS (http://www.esri.com/software/arcgis/arcgis-for-desktop) and SigmaPlot (http://www.sigmaplot.com/).

Filtered GWD rates (GWD_a_) were calculated using constrained forward modeling based on filtered GRACE TWS changes minus filtered SMS changes from GLDAS-1 Noah. GWD_a_ rates with a mean of 1.9 cm/a represent what GRACE can see after low-pass filtering for removing high-frequency noise (Fig. 3c). Therefore, directly using GWD_a_ rates from GRACE would result in significantly reduced GWD rates compared with the ‘realistic’ GWD rates (Fig. 3b). When the GWD_a_ rates from GRACE and the filtered GWD_s_ rates are the same (Fig. 3c), the forward modeling solution has converged and is terminated with the last updated GWD_s_ rates taken as the resulting GWD rates using this approach.

Interannual variability in surface water is assumed to be negligible in the calculation of GWD_a_, which has also been assumed in previous studies for this region^16^,^21^ and demonstrated by WGHM in our study (see Supporting Information 3 and Fig. S17). In addition, both globally (Fig. 2c) and regionally (Fig. S18b) constrained forward modeling resulted in a reasonable distribution of GWD compared with the GWD pattern from PCR-GLOBWB. However, constrained forward modeling generated lower regionally averaged GWD rates (3.1 cm/a for globally constrained and 3.1 cm/a for regionally constrained) than the GWD rate of 6.7 cm/a from PCR-GLOBWB. Locally constrained forward modeling, again, resulted in an unrealistic spatial pattern and magnitude of spatially averaged GWD rates (not shown) due to the assumption of GWD restricted to the three-state region.

Interestingly, unconstrained forward modeling can also provide a similar magnitude of GWD of ~3.0 cm/a compared with the result of ~3.1 cm/a from globally and regionally constrained forward modeling. However, it is apparent that the GWD spatial distribution from unconstrained forward modeling does not seem reasonable relative to the GWD pattern from PCR-GLOBWB and groundwater-level data. This is determined by the intrinsic characteristic of unconstrained forward modeling that no a priori information is used to constrain the recovered signal distribution. However, it could recover the magnitude of filtered signals as long as the boundary covers all areas with signal leakage. This is also the reason in *Chen et al*.^21^ why a broader area than the three-state region was prescribed to account for all recovered GWD using unconstrained forward modeling. From the numerical simulations using hypothetical data and the simulations using synthetic data from PCR-GLOBWB, it has been shown that signal interference from the region surrounding the three-state region could result in a marked reduction in signal leakage from the three-state region. Therefore, the overall effectiveness of unconstrained forward modeling to recover signal loss for the three-state region is a particular case and may not be generalized to other regions. Recovering signal loss using unconstrained forward modeling depends on the relative difference in signal variation within and outside a study region of interest.

### Comparison of GRACE-based estimates with groundwater-level data

Filtered and original (unfiltered) SMS anomalies from three LSMs (i.e., GLDAS-1 Noah, Mosaic, and VIC) are consistent (see shading areas representing one standard deviation of the three SMS anomaly time series) (Fig. 3d). The mean of the three time series shows a slightly decreasing trend from Jan 2003 to the summer in 2010 and an appreciable recovery from drought in Southeast and South Asia since then^41^. Furthermore, filtered SMS anomalies from these LSMs show slightly higher amplitudes than the original time series, indicating signal amplification from the area surrounding the three-state region during low-pass filtering.

It is apparent that the filtered TWS anomalies have larger seasonal amplitudes and long-term trends than SMS and GWS anomalies (Fig. 3e), which is reasonable given that TWS changes integrate surface and subsurface water storage changes. The filtered GWS anomalies show a steady decreasing trend from 2003 to the summer of 2010, but the seasonal amplitudes of filtered GWS anomalies are small, resulting from different phases and uncertainties from TWS and SMS anomalies (more noises). In addition, TWS and SMS anomalies have a similar phase of variations, but the filtered GWS anomalies show 1–2 month time lag compared with the TWS and SMS anomalies. This is reasonable because GWS variations should lag climatic variations relative to TWS and SMS and should also include impacts (phase) from human activities (e.g., intensive irrigation with groundwater). Therefore, both the seasonal amplitude and long-term trend in GWS anomalies are quite different from TWS and SMS anomalies. Note that our purpose is to recover long-term trends in GWS time series using different approaches. The filtered GWS anomalies are just an intermediate variable that essentially reveals temporal variability in GWS. For obtaining a precise magnitude of changes in GWS, signal losses in filtered GWS anomalies should be restored especially for long-term trends first before interpreting GWS changes over this region.

In general, GWS anomaly time series from four approaches show steady decreasing trends from Jan 2003 to Jun 2010. Since then, all GWS time series tend to recover by the end of 2011. GWS started to decline from 2012 onwards by the end of the study period Jul 2013 (Fig. 3f). However, different approaches for generating GWS anomalies can differ in seasonal amplitude. The GWS anomalies from the multiplicative approach show relatively higher seasonal amplitudes than the other estimates, with a multiplicative factor of 2.1 derived from the amplitude ratio of the basin function and filtered basin function (Fig. S3a,c), similar to ~1.95 derived by the previous study^16^. The multiplicative factor that assumes a uniform distribution within a study region but no variations outside the region resulted in both the highest seasonal variability and GWD rate. In contrast, the scaling factor of 0.67 derived from CLM4.0 provided by the JPL website resulted in the lowest seasonal amplitudes in GWS anomaly. The highest differences between the two time series occurred in wet and dry seasons during the study period, e.g., ~150 mm in Sep 2003 (the wet case) and ~130 mm in Jun 2010 (the dry case) at the end of the extreme drought in Southeast Asia in spring 2010.

GWS anomalies from the forward modeling approach show appreciably larger seasonal amplitudes than the additive correction approach. Scaling factors derived from three GLDAS-1 LSMs for the three-state region are all less than 1 (0.92, 0.79, and 0.75 for Noah, Mosaic, and VIC, respectively, Fig. 3d), indicating that signals for seasonal variability in water storage from GRACE tend to be amplified due to stronger signals from the surrounding areas during low-pass filtering. Therefore, a scaling factor less than 1 needs to be applied to reduce the leakage error. CLM4.0 provided a markedly lower scaling factor (0.67) than other LSMs examined, thereby reducing seasonal signals in TWS change significantly.

Long-term trends in GWS further reflect appreciable differences in signal restoration from different approaches. Trends in GWS change for the periods 2003–2010, 2005–2010, and 2003–2012 using linear regression without/with GWS errors time series were calculated (Table 1 and Methods section). For the period Jan 2003 through Dec 2010, the multiplicative and the scaling factor approaches generated the highest (3.8 ± 0.2 cm/a) and lowest (0.9 ± 0.2 cm/a) GWD rates among the four approaches. The multiplicative approach amplified both seasonal amplitudes (see Jun 2010) and long-term trends. The lower estimates of GWS changes from the scaling factor approach resulted mainly from the low scaling factor of 0.67 from CLM4.0, which dampened both seasonal amplitudes and secular changes (Fig. 3f). The forward modeling approach provided estimates of GWS change rates less (3.1 ± 0.1 cm/a) than the multiplicative approach but greater than both the scaling factor and additive (1.9 ± 0.3 cm/a) approaches. Similar results were also found for the period Jan 2005–Dec 2012 and Jan 2003–Dec 2012 (Supporting Information 4). The forward modeled GWD (3.1 ± 0.1 cm/a) in this study agrees well with the GWD rate (2.8 cm/a) from the *in situ* groundwater-level measurements and specific yield. The multiplicative approach and PCR-GLOBWB appeared to overestimate GWD in this region. The scaling factor and additive approaches, however, appeared to underestimate GWD rates.

## Discussion

Our estimate of GWD from constrained forward modeling for the three-state region during the five-year period Aug 2003 through Oct 2008 is 2.5 ± 0.1 cm/a, which is less than the estimate of 4 ± 1 cm/a for the period Aug 2002 through Oct 2008 by *Rodell et al*.^16^. One of the possible reasons for a higher estimate for the GWD rate from *Rodell et al*.^16^ lies in the assumption inherent in the multiplicative approach that all GWD was restricted to the three-state region, i.e., a binary averaging kernel was assumed to be 1 in the three-state region but 0 outside the region. This assumption does not seem reasonable considering the spatial patterns of GWD from PCR-GLOBWB and groundwater-level measurements examined in this study. The surrounding areas of the three-state region, e.g., Pakistan and western Uttar Pradesh, exhibit significant GWD. Assuming no GWS amplitude outside the averaging kernel would result in an overestimate of the multiplicative factor and consequently overestimation of both the seasonal cycle and secular change in GWD. In addition, our estimate of the GWD rate (3.1 ± 0.1 cm/a) for the three-state region during the period Jan 2003–Dec 2010 is appreciably higher than the mass loss estimates of *Jacob et al*.^29^ of 1.9 cm/a (35 km^3^/a) over North India for the period Jan 2003–Dec 2010 using 350 km Gaussian filtering that was attributed primarily to GWD in the region. The reason could be the use of the mascon solution in *Jacob et al*.^29^ that covers a broader area than the three-state region, reducing the mass loss rate.

The constrained forward modeling-based GWD rate during the period Jan 2003–Dec 2012 in our study was 2.1 ± 0.1 cm/a for the three-state region, which is consistent with what *Chen et al*.^21^ obtained using unconstrained forward modeling. This has been demonstrated by numerical simulation using synthetic data from PCR-GLOBWB that constrained and unconstrained forward modeling can generate a similar magnitude of GWD due to the spatial distribution of GWD within and outside the three-state region. Furthermore, our GWD estimates using constrained forward modeling make GRACE estimates more meaningful by incorporating the a priori information on groundwater-based irrigation. The limitations of unconstrained forward modeling without exact spatial pattern of GWD and requiring post-processing of manually defining the extent of GWD were greatly reduced. The footprint for GRACE data is large (e.g., 200,000 km^2^). Compared with traditional processing that can only provide basin-scale GWD, constrained forward modeling makes use of the GWD pattern from a priori knowledge, which is extremely important to assess the local vulnerability of water supply especially during drought^5^. As the quality of GRACE data and related processing techniques improve, GRACE satellites will be more valuable in assessing GWD over aquifers globally, which should be important for evaluating and improving the sustainability of agriculture and water resource management.

GRACE signal restoration for aquifers is challenging because GWS changes may have different phases of seasonal and interannual variability resulting from climate variability and human activities. Scaling factors for correcting for seasonal cycles and secular changes of GWS should be applied separately^16^,^33^. Note that SMS may not show marked interannual variability due to long-term intensive irrigation that results in moist soils most of time (referring to Fig. S17a). LSMs/GHMs are powerful tools for depicting seasonal cycles and therefore generating scaling factors for correcting for seasonal variability in SMS or GWS. Surface water storage over this region does not seem to have significant interannual variability, either. Therefore, the key issue to estimate GWS changes from GRACE satellites is to compute scaling factors for correcting for secular changes in TWS or directly compute secular changes in GWS as forward modeling does.

Use of a single scaling factor from LSMs to correct for both seasonal cycles and secular changes may be highly uncertain. Our study showed the lowest estimate of the GWD rate of ~1 cm/a for the period 2003–2010 due to a lower scaling factor of 0.67 from CLM4.0. This seems to be unreasonable and much lower than those estimated by previous studies and groundwater-level monitoring data. GWD rates from the additive approach were higher than those for the scaling factor approach, but lower than those from the forward modeling and multiplicative approaches. This can be explained by the way in which bias and leakage effects were corrected depending on GLDAS-1 Noah output. Because Noah does not have a groundwater component, the bias computed using Noah-based SMS output may be less than what it should be, resulting in relatively lower estimates of seasonal amplitudes and secular changes in TWS. Therefore, one of the most important limitations in scaling factor and additive approaches rests with the dependence on LSMs that do not account for the impact of anthropogenic GWS changes, though some LSMs accommodate the effect of natural variability in GWS (e.g., CLM4.0). Both constrained and unconstrained forward modeling presented in this study can provide reasonable estimates of GWD rates relative to groundwater-level data. The limitation of constrained forward modeling is that it may not completely recover the spatial pattern of signals over regions with highly heterogeneous mass changes. We suggest that each approach has advantages and limitations. The range of GWD estimates is valuable in understanding uncertainties in GWD estimates for aquifers, especially when GRACE-based monitoring is taken as an independent approach to evaluate model output. This study highlights the importance of selecting appropriate processing strategies for restoring GRACE signals and uncertainties in GWD estimation for aquifers from different approaches. Constrained forward modeling with a priori spatial distribution of GWD from PCR-GLOBWB or ground-based measurements is useful in recovering both the magnitude and distribution of GWD rates from GRACE satellites for the Northwest India Aquifer.

## Methods

### Constrained and unconstrained forward modeling

Constrained forward modeling makes use of a priori information regarding the spatial distribution of signals whereas unconstrained forward modeling does not. The basic idea of forward modeling is to iteratively adjust the filtered signal (and distribution for constrained forward modeling) to numerically (and spatially) approach a reference. The reference is often from filtered GRACE mass change rates (and synthetic data from GHMs or ground-based observations). An illustration of unconstrained and constrained forward modeling of GWD rates from GRACE is given below (also referring to Fig. S19a).

First, SH solutions of CSR RL05 and GLDAS Noah soil moisture were destriped^17^,^31^ (not for GLDAS data), truncated at the maximum degree and order of 60, and filtered using a 300 km Gaussian filter^17^. The first-order estimate of GWS change rates can be derived by the least square fit for the filtered GWS anomalies from filtered GRACE TWS minus filtered GLDAS SMS anomalies. The first-order estimate of GWS change rates is termed the apparent GWD rate (GWD_a_), which is the incomplete estimate of GWD rates whose signals have been dampened during the low-pass filtering process. Second, a dummy GWD distribution is created, with its initial values taken as GWD_a_ or random for each grid cells, termed the simulated GWD rate (GWD_s_). Third, the difference between the filtered GWD_s_ and GWD_a_ is added back to GWD_s_ until the filtered GWD_s_ numerically converges to GWD_a_. Under the condition that the filtered GWD_s_ and GWD_a_ are equal or less than a threshold, the last update of GWD_s_ is then taken as the final estimate of GWD through unconstrained forward modeling. For constrained forward modeling, only the differences between filtered GWD_s_ and GWD_a_ for constrained areas (often from synthetic data used as a priori knowledge) are added back to GWD_s_. Note that GWD_s_ needs to be continually updated during the iterative process, but GWD_a_ is determined by GRACE data that does not change.

We hypothesize that the spatial distribution of original signals may affect the use of constrained and unconstrained forward modeling. In this study, both constrained and unconstrained forward modeling were first evaluated using: (1) hypothetical data with varying configurations of uniform and heterogeneous signal distributions, and (2) synthetic data of GWD from PCR-GLOBWB. A flowchart for the two tasks is shown in Fig. S19b. Based on this, GWD for the three-state region and surrounding areas from GRACE satellites was estimated using both unconstrained and constrained forward modeling.

### Reconstruction of groundwater storage change time series

Reconstruction of GWS time series is performed in two steps. The first step is to derive long-term trends from forward modeling illustrated in the above section. The second step is to reduce the bias and leakage effects for seasonal variations using scaling factors derived from LSMs/GHMs^16^,^33^. The assumptions involved in this reconstruction approach are: (1) LSMs reliably depict seasonal variations in GWS changes; and (2) seasonal and long-term changes can be recovered separately:


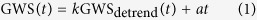


where GWS(*t*) is the corrected GWS anomaly time series for aquifers as a function of time (*t*); GWS_detrend_(*t*) is the seasonal cycle of the filtered GWS anomaly time series from GRACE data in which the incomplete long-term trends are removed; *k* is the scaling factor derived from filtered and unfiltered LSM/GHM output. In this study, we used PCR-GLOBWB output^12^,^42^,^43^ to create scaling factors for correcting for seasonal cycles of GWS changes; and *a* is the long-term change rate of GWS through forward modeling illustrated in the above section.

Use of LSMs to quantify long-term trends in water storage components may have large uncertainties because LSMs do not simulate all the components of the water budget (e.g., GWS)^33^. However, LSMs may still be useful in quantifying relative patterns of water storage changes within and outside an area (aquifer) at the seasonal scale. The forward modeled GWS rates will subsequently be added back to the seasonal cycle of GWS anomalies (equation (1)) from filtered GRACE GWS anomalies with long-term trends removed first and bias and leakage effects corrected using scaling factors from LSMs. The reconstruction approach can also be applied in a piecewise manner. This means that forward modeling is performed for each sub-period with a statistically significant trend due to climate variability and/or anthropogenic activities. The resulting GWS rates will be added back to the seasonal cycle of GWS anomalies for each sub-period to reconstruct the monthly GWS anomaly time series that retains both the seasonal cycle with bias and leakage effects corrected by a scaling factor from LSMs and the secular change derived from forward modeling.

### Study strategies

First, a range of numerical simulations using hypothetical data was performed to evaluate the utility of unconstrained and constrained forward modeling to recover signal loss due to low-pass filtering. Five configurations of signal patterns were tested, with the aim of obtaining a comprehensive understanding of how different patterns of original data impact the ability of these forward modeling techniques to recover signal loss, including: (1) assigning a uniform GWD rate of −50 mm/a across the study region, (2) assigning random GWD rates ranging from 0 to −100 mm/a across the study region, (3) assigning a uniform GWD rate of −50 mm/a for the right half of the study region but random GWD rates ranging from 0 to −100 mm/a for the left half; (4) assigning a uniform GWD rate of −50 mm/a for the lower half but random GWD rates ranging from 0 to −100 mm/a for the upper half, and (5) assigning a uniform GWD rate of −50 mm/a for a circular area in the central half of the study region but random GWD rates ranging from 0 to −100 mm/a for its surroundings within the study region.

Second, synthetic data were used to demonstrate how signals vary during low-pass filtering and how the filtered signal is recovered using forward modeling in a more realistic environment. The modeled data are often obtained from GHMs/LSMs. Here, the utility of both unconstrained and constrained forward modeling for the three-state region was tested using GWD estimates from PCR-GLOBWB. We suggest that in practice there are three types of constrained forward modeling. The first type makes use of the global distribution of GWD simulations from PCR-GLOBWB, termed globally constrained forward modeling. Note that globally constrained forward modeling may suffer from uncertainties due to noise in GRACE signals. The second type only uses a broader area of the spatial pattern of synthetic data that includes the surroundings of a study region where significant signal variations outside the study region may occur, termed regionally constrained forward modeling. The third type makes use of spatial patterns of signal variations from models or observations completely restricted to a study region, termed locally constrained forward modeling.

Third, GWD rates for the three-state region during the periods 2003–2010, 2005–2010 and 2013–2012 were estimated using GRACE and GLDAS-1 LSM SMS changes within the framework of constrained and unconstrained forward modeling. The GWD distribution from PCR-GLOBWB was taken as a priori information for implementing constrained forward modeling. Following the three parts of work mentioned above, GWS anomaly time series from forward modeling were reconstructed and compared with those from traditional approaches, i.e., the single scaling factor approach, additive approach, and multiplicative approach. Our estimates of GWD for the three-state region were further compared with groundwater-level monitoring data and published studies, with advantages and limitations of different approaches for aquifers being systematically examined.

### Error analysis

We derived error time series for GWS anomalies and subsequently performed weighted least square regression. Errors in GWS time series were computed by root mean square of errors of TWS changes and SMS changes^17^ at the monthly scale. Errors in TWS changes include GRACE L1 measurement errors and leakage errors. The measurement errors were quantified by looking at the standard deviation of TWS changes over ocean for the same latitude of a study basin^44^, and the leakage errors were quantified by different LSMs to approximate TWS changes for a study region. Errors of SMS changes were quantified by the standard deviation of SMS from four GLDAS-1 LSMs and two GHMs (see Table S2)^17^.

## Additional Information

**How to cite this article**: Long, D. *et al*. Have GRACE satellites overestimated groundwater depletion in the Northwest India Aquifer? *Sci. Rep*. **6**, 24398; doi: 10.1038/srep24398 (2016).

## Supplementary Material

Supplementary Information

## Figures and Tables

**Table 1:** GWD rates (cm/a) derived from GRACE GWS changes from four approaches examined in the three-state region (Punjab, Haryana & Deli, and Rajasthan) of Northwest India for three periods.

Study period	Regression approach	Scaling factor (CLM4.5)	Additive correction	Multiplicative correction	Forward Modeling
Jan 2003–Dec 2010	w/o error	0.9 ± 0.2	1.9 ± 0.3	3.8 ± 0.2	3.1 ± 0.1
	w/ error	0.9 ± 0.2	1.9 ± 0.2	3.5 ± 0.3	3.0 ± 0.1
Jan 2005–Dec 2010	w/o error	1.0 ± 0.3	1.8 ± 0.4	3.8 ± 0.4	3.1 ± 0.1
	w/ error	0.9 ± 0.2	1.8 ± 0.3	3.4 ± 0.4	3.0 ± 0.1
Jan 2003–De 2012	w/o error	0.6 ± 0.1	1.1 ± 0.2	2.5 ± 0.2	2.1 ± 0.1
	w/ error	0.7 ± 0.1	1.2 ± 0.2	2.4 ± 0.2	1.9 ± 0.1

‘w/o error’ means that GWS errors were not considered in the linear regression analysis. ‘w/ error’ means that errors in GWS estimation were considered using weighted linear least squares regression, expressed as the inverse of squared errors in the weighting process.
