# Supplementary material for: Have GRACE satellites overestimated groundwater depletion in the Northwest India Aquifer?
Source: Sci Rep. 2016 Apr 14;6:24398. doi: 10.1038/srep24398 (PMC4830960; doi:10.1038/srep24398)
Supplement: Supplementary Information [file srep24398-s1.pdf]

Have GRACE satellites overestimated groundwater storage depletion in the Northwest India Aquifer? Supporting Information

Di Long[1], Xi Chen[1], Bridget R. Scanlon[2], Yoshihide Wada[3,4,5,6], Yang Hong[1,7], Vijay P. Singh[8,9], Yaning Chen[10], Cunguang Wang[1], Zhongying Han[1], and Wenting Yang[1]

1. State Key Laboratory of Hydroscience and Engineering, Department of Hydraulic Engineering, Tsinghua University, Beijing 100084, China (Email: dlong@tsinghua.edu.cn)

2. Bureau of Economic Geology, Jackson School of Geosciences, The University of Texas at Austin, Austin, TX 78758, United States

3. Department of Physical Geography, Utrecht University, Utrecht, Netherlands

4. NASA Goddard Institute for Space Studies, New York, NY 10025, United States

5. Center for Climate Systems Research, Columbia University, New York, NY, United States

6. International Institute for Applied Systems Analysis, Laxenburg, Austria

7. Department of Civil Engineering and Environmental Science, University of Oklahoma, Norman, OK 73072, United States

8. Department of Biological & Agricultural Engineering, Texas A&M University, College Station, TX 77843, United States

9. Department of Civil & Environmental Engineering, Texas A&M University, College Station, TX 77843, United States

10. State Key Laboratory of Desert and Oasis Ecology, Xinjiang Institute of Ecology and Geography, Chinese Academy of Sciences, Urumqi, Xinjiang 830011, China

The supplementary material file includes:

Section 1: Typical approaches of restoring filtered GRACE signals

Section 2: Study region and data

Section 3: Surface water storage changes from WGHM2.2 standard version

Section 4: Comparison of different approaches for deriving GWS changes for Jan 2003-Dec 2012

Supplementary Figures S1-S19

Supplementary Tables S1-S2

1. Typical approaches of restoring filtered GRACE signals

Two types of error need to be corrected properly during the GRACE signal restoration process, i.e., the bias and leakage errors[1]. First, signal loss within a study area of interest due to a filtered averaging kernel (or basin function) that defines the area is termed the bias effect. Second, signal gain mostly from the surrounding region can contaminate the signal within the study basin, which is termed the leakage effect. In some literature, bias and leakage are not discriminated; both are termed the leakage effect. For a more precise discussion, we make a difference between the bias and leakage effects in this study following the study[1]. Typical approaches for correcting for the bias and leakage effects can be categorized as (1) approaches using water storage changes from LSMs, and (2) approaches depending less on water storage changes from LSMs. Approaches using water storage estimates from LSMs are the scaling factor and additive correction approaches. For the scaling factor approach, LSM output undergoes the same low-pass filtering as applied to GRACE data. The filtered and unfiltered water storage changes from LSMs are subsequently compared to generate scaling factors using the least square fit to restore filtered GRACE TWS changes[2]. The additive correction approach takes advantage of water storage changes from LSMs to compute bias and leakage separately, and therefore the filtered GRACE TWS changes can be restored by adding the bias and removing the leakage estimates[1,3].

It is challenging to perform signal restoration for aquifers using approaches that depend largely on LSM output, because most LSMs do not simulate GWS changes induced by groundwater pumping. Global hydrological models (GHMs) accommodate the impact of human activities on surface and subsurface water, e.g., WaterGap Global Hydrological Model (WGHM) and PCR-GLOBWB. However, these models cannot simulate lateral flow that would increase groundwater recharge in piedmont areas, and therefore overestimate GWD, e.g., the North China Plain[4]. Implementation of LSMs/GHMs often requires tremendous efforts in data collection and processing, greatly reducing their operability in groundwater resource management. Investigating and developing algorithms to restore filtered GRACE signals depending less on LSMs/GHMs are therefore extremely important to estimate GWD in aquifers.

Examples of such approaches include the multiplicative correction approach[5-7] and unconstrained and constrained forward modeling[8,9].

The multiplicative correction approach, as was applied in *Rodell et al*.[10], assumes that TWS or GWS changes are uniformly distributed within a study aquifer, and mass changes surrounding the basin are assumed to be zero or can be estimated by LSM output[1,11-13]. Seasonal amplitudes and long-term trends in TWS or GWS can be recovered using a multiplicative factor from a filtered normalized averaging kernel (or basin function) and/or depending partially on LSMs for correcting for the leakage effect. Unconstrained or constrained forward modeling that mimics the low-pass filtering and corrects for the signal loss through an iterative approach has been shown to be effective in reducing the leakage effect over the cryosphere, e.g., Antarctic[8]. There are two types of forward modeling techniques[9,14]. One is unconstrained forward modeling, as used in *Chen et al*.[9] to estimate GWD in the NWIA, that performs iterative correction without using a priori knowledge about the spatial distribution of signal variations. The other one is constrained forward modeling that uses a priori information regarding the spatial distribution of water storage changes to constrain the recovered signals. Unconstrained forward modeling does not provide detailed spatial distribution of forward modeled GWD, and the leakage effect cannot be completely recovered for a specific region of interest. Constrained forward modeling was applied to simulate mass changes over Antarctic[8], in which a uniform distribution of mass changes was allocated to their study regions. This simplification may hamper a further understanding of the utility of constrained forward modeling to recover signals.

2. Study region and data

2.1 Northwest India Aquifer

We use the three-state region (i.e., Punjab, Haryana & Delhi, and Rajasthan with a total area of 438,296 km^2) in the NWIA as a test-bed to compare different approaches for deriving GWS anomaly time series

from GRACE satellites (i.e., forward modeling, scaling factor, additive correction, and multiplicative correction), the same as *Rodell et al*.[10]. A comprehensive description of scaling factor, additive correction, and multiplicative correction approaches can be found in the reference[15]. India has the highest national groundwater extraction rate in the world, accounting for one fifth of the global total[16]. The NWIA is a vast agricultural region that relies heavily on groundwater withdrawals for its irrigation water supply[17], and has been undergoing the highest GWD among aquifers globally due to irrigation either through GRACE satellite estimates[10] or GHM modeling[4]. In this study, we focused on the three-state region that includes Punjab, Haryana & Delhi, and Rajasthan (Figure 1) with an area of 436,390 km^2 examined in *Rodell et al*.[10] and that was undergoing intense GWD reported by the India Ministry of Water Resources. The GWD in these states has already exceeded the unsafe groundwater development stage.

The study region is intensively irrigated with conjunctive use of surface water and groundwater. There is ~30% of the study region equipped for irrigation (Figure 1 (a)) according to AQUASTAT data of the Food and Agricultural Organization (FAO) of the United Nations, in which 74% of the equipped area is irrigated with groundwater (Figure 1 (b)) and the left with surface water. Land cover data in 2005 and 2009 with a spatial resolution of 300 m from the European Space Agency GlobCover Portal indicate that the north and northeast of the three-state region were dominated by irrigated croplands (44% - 48% of the total study area, Figure S15). However, the west and southwest regions were dominated by mosaic vegetation/croplands (10% - 13%), bare areas (8% - 11%), and mosaic grassland/forest-shrubland (10%). Only 16% of Rajasthan state was irrigated. GWD may occur in irrigated areas but not all irrigated areas would exhibit GWD due to conjunctive use of surface and groundwater. Furthermore, areas without irrigation infrastructure (e.g., west Rajasthan) will not likely show GWD, which will be demonstrated using groundwater-level data and a GHM in the Results Section.

2.2 Data

Data used in this study include GRACE SH coefficients (CSR RL05) (ftp://podaac.jpl.nasa.gov/allData/grace/L2/CSR/RL05/) and monthly soil moisture and snow water equivalent from three four LSMs (Noah, Mosaic, and VIC) in GLDAS-1 at a spatial resolution of $0.25°\times0.25°$ (Noah) or $1°\times1°$ (Mosaic and VIC). Scaling factors from CLM4.0 provided by the JPL website (http://grace.jpl.nasa.gov/data/gracemonthlymassgridsland/) were also used to restore filtered GRACE signal by the scaling factor approach[2]. Monthly GWD and GWS changes at the spatial resolution of $0.5°\times0.5°$ from PCR-GLOBWB for the period 2003-2010 were used for testing constrained forward modeling, and to create scaling factors for correcting for seasonal variations in GWS changes over this region. TWS, SWS, SMS, and GWS changes from the WaterGAP WGHM2.2 model[18] for the period 2003-2009 were obtained for use in checking the spatial pattern of GWD from PCR-GLOBWB and examining interannual variability in SWS. Descriptions about characteristics of LSMs and GHMs used in this study and associated advantages and disadvantages are provided in Table S2.

Groundwater levels (i.e., minus groundwater depth to the surface) in districts of the three-state region and its surroundings for the period 2005-2010 monitored by India's Central Ground Water Board (CGWB) were obtained from the Groundwater Information System (http://gis2.nic.in/cgwb/Gemsdata.aspx) hosted by the National Informatics Centre. There are multiple groundwater-level monitoring sites for each district, but the exact coordinate information for each well is not available. Therefore, GWD rates from groundwater-level monitoring data were estimated at the district scale. For most sites, there are four groundwater-level measurements in each year.

Groundwater-level measurements lower than 5% percentile or higher than 95% percentile of the frequency distribution of the measurements within a district in a year were precluded from the analysis to reduce outliers and part of the impact of confined aquifers. Around 2000 sites (the number of sites slightly varies with year) in the three-state region were finally used for GWD estimation. The averaged groundwater-level from the selected sites in a district for each year was computed to approximate the

annual groundwater-level for a district. Linear regression was subsequently performed for each district-scale groundwater-level annual time series to derive the slope of groundwater-level variations. Recommended specific yield values for each type of geological formations in India were obtained from the report[19]. Fractions of geological formations for each state were obtained from the report[20]. The specific yield for a state was therefore taken as the area-weighting specific yield according to the fractions of each geological formation in a state and its associated specific yield values. By multiplying water-level time series for a district with the specific yield of the state associated with the district, the ground-based GWD rate can be derived and taken as reference for evaluating GWD from GRACE using different approaches.

3. Surface water storage changes from WGHM2.2 standard version

To examine if there is interannual variability in surface water storage (including reservoir storage changes, river storage changes, and lake and wetland storage changes) and soil moisture, we further looked at SWS and SMS changes from WGHM2.2 which comprehensively considers SWS, SMS, GWS changes and human impact on water storage changes. WaterGAP[21] consists of both the WGHM model[22] and five water use models for the following sectors: irrigation[23] and livestock, household, manufacturing and cooling of thermal power plants[24]. WGHM computes time series of rapid-surface and subsurface runoff, groundwater recharge and river discharge as well as water storage variations in canopy, snow, soil, groundwater, lakes, man-made reservoirs, wetlands and rivers as a function of climate, soil, land cover, relief and observed river discharge. Location and size of lakes, reservoirs, and wetlands are defined using the global lakes and wetland database (GLWD)[25], with an addition of more than 6000 man-made reservoirs[22]. Groundwater storage is affected by diffuse groundwater recharge via the soil, which is modeled as a function of total runoff, relief, soil texture, hydrogeology and the existence of permafrost or glaciers. Focused groundwater recharge from rivers, lakes, and wetlands is not simulated in WGHM. This type of recharge may be important, in particular in semi-arid and arid regions.

Sectorial water uses for irrigation livestock, households, manufacturing and cooling of thermal power plants are computed using separate models. The irrigation model GIM[23] computes consumptive water use, i.e., the part of the withdrawn water that evaporates during use. For all other sectors, both water withdrawal and consumptive water use are quantified by the water use models. Taking into account information on the water source, and making assumptions about irrigation water use efficiencies and return flows, the sub-model GWSWUSE computes net abstractions from groundwater and from surface water[26]. Net abstractions are computed as the difference between water withdrawals from the specific source and the return flows from water use to the source. Net abstractions are negative if abstractions (withdrawals) are less than return flows, which can only occur in the case of irrigation from surface water. In this comparison, we assume that WGHM water storage changes represent reality because the WGHM model comprehensively considers water storage changes in surface water, soil layers, and aquifers, and especially accommodates human impacts on SWS and GWS changes.

SWS and SMS changes from WGHM2.2 clearly show that there is no measurable interannual variability in the two components of TWS. However, GWS shows a steady decreasing trend since 2003 onwards (Figure S17a). This further demonstrates that interannual variability in SWS and SMS is not significant and therefore TWS changes from GRACE and LSM/GHM are primarily controlled by GWS changes induced by groundwater abstractions for irrigation.

4. Comparison of different approaches for deriving GWS changes for Jan 2003-Dec 2012

Similar results were also found for the period Jan 2003-Dec 2012 that the multiplicative and the scaling factor approaches generated the highest (-2.4±0.2 cm/a) and lowest (-0.7±0.1 cm/a) GWS change rates, respectively. The forward modeling approach-based GWS rate (-1.9±0.1 cm/a) followed that from the multiplicative factor approach. Again, the additive correction approach provided the GWS change rate (-1.2±0.2 cm/a) slightly higher than that from the scaling factor approach (-0.7±0.1 cm/a). The relatively lower estimates of secular changes in GWS from the additive correction can be attributed to the bias and

leakage corrections using TWS estimates from the GLDAS-1 Noah model. Noah does not account for appreciable changes in GWS over this region, resulting in a dampened signal for bias computation and therefore relatively lower seasonal amplitudes and secular changes in GWS. As is also indicated by Landerer and Swenson[2], applying a single scaling factor would not properly correct for the seasonal amplitudes and secular changes in TWS at the same time. Therefore, results from the scaling factor and multiplicative approaches provide the extreme estimates (the highest and lowest) of both seasonal amplitudes and secular changes as shown in our study. The forward modeling-based GWS anomalies appear to be more reasonable based on groundwater-level monitoring data.

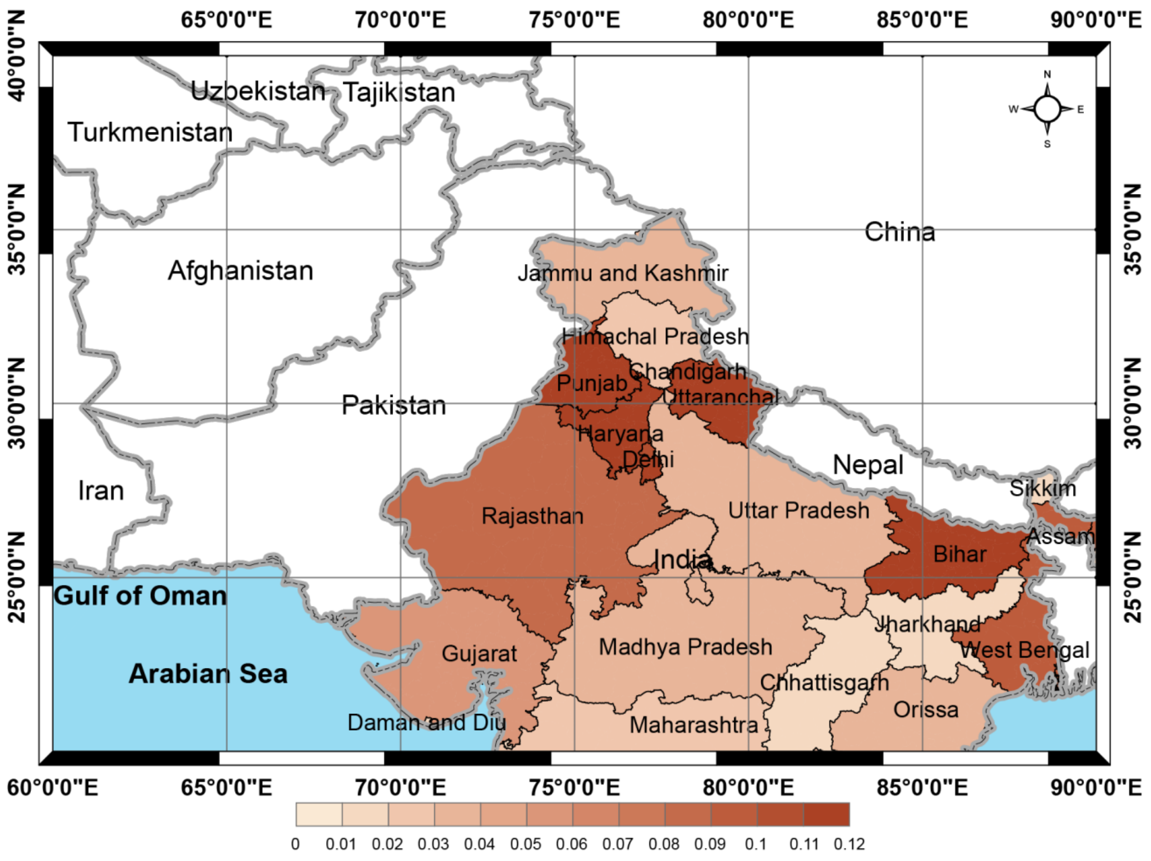

Figure S1 Specific yield over the three-state region and its surroundings. Map was created using ArcGIS (http://www.esri.com/software/arcgis/arcgis-for-desktop).

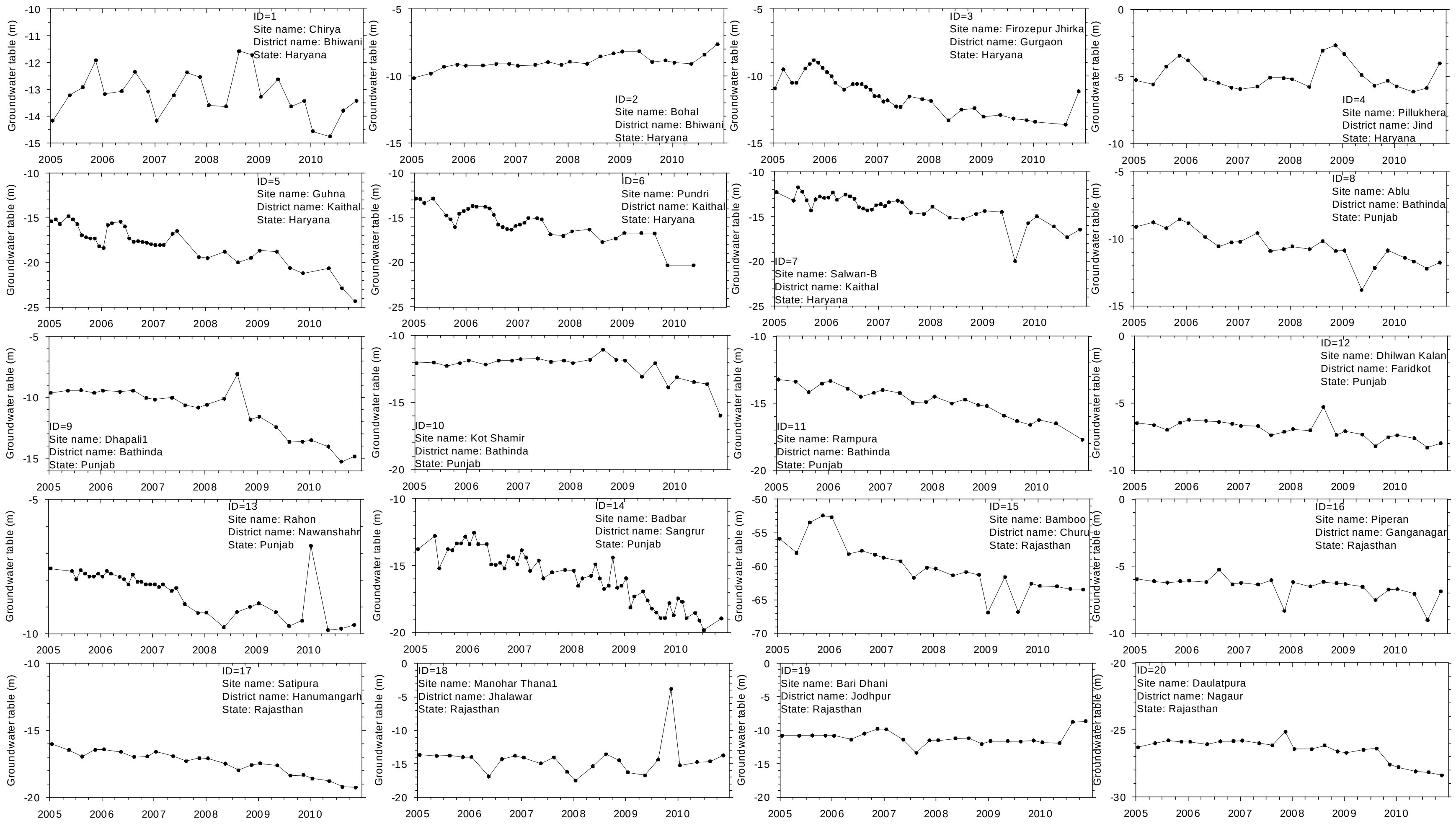

Figure S2 Groundwater-level measurements for 20 selected sites in the three-state region. Coordinates of the 20 sites shown in open circles in Figure 1 (c) were derived from Google Maps according to the site name, district name, and state. Map was created using SigmaPlot (http://www.sigmaplot.com/).

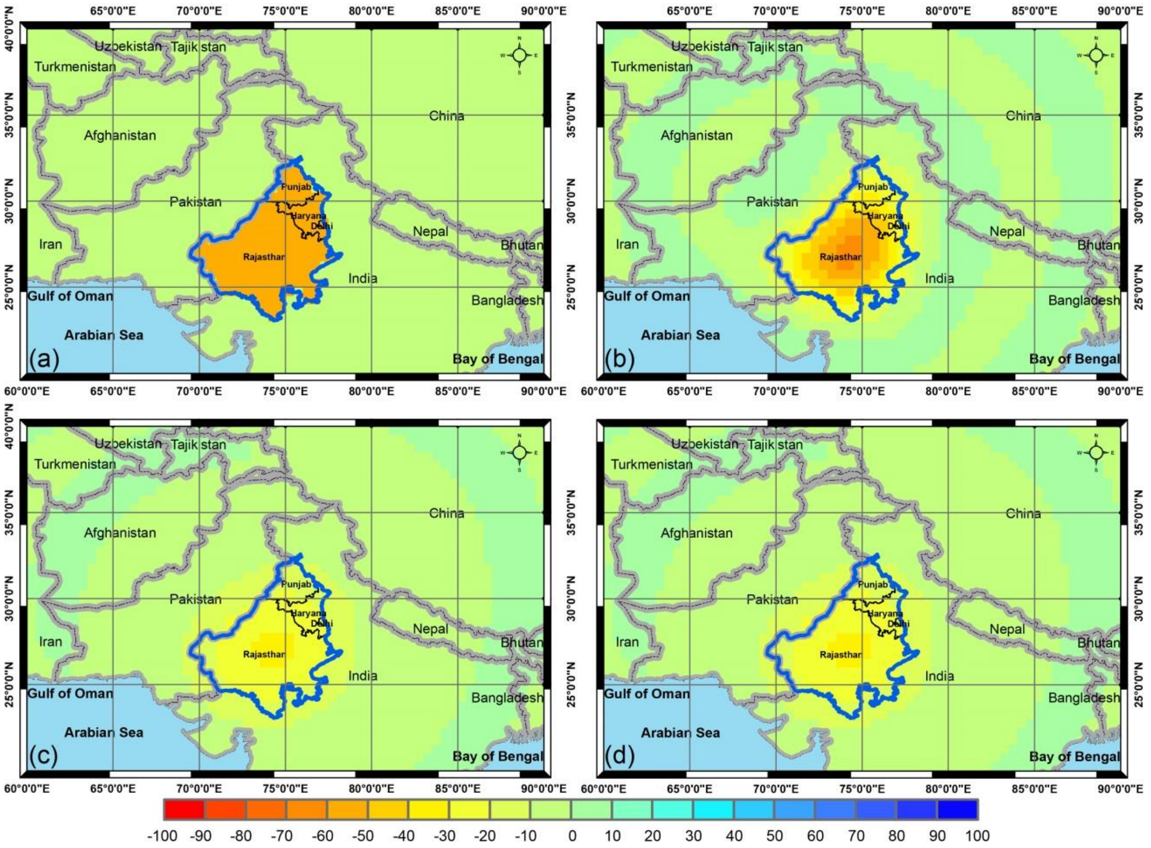

Figure S3 Evaluation of unconstrained forward modeling using (a) a hypothetic uniformly distributed GWD rate with a spatial mean of -50 mm/a, (b) forward modeled GWDs rate distribution after 500 iterations, (c) GWDa derived from (a) after low-pass filtering, and (d) filtered GWDs derived from (b) after low-pass filtering. Map was created using ArcGIS (http://www.esri.com/software/arcgis/arcgis-for-desktop).

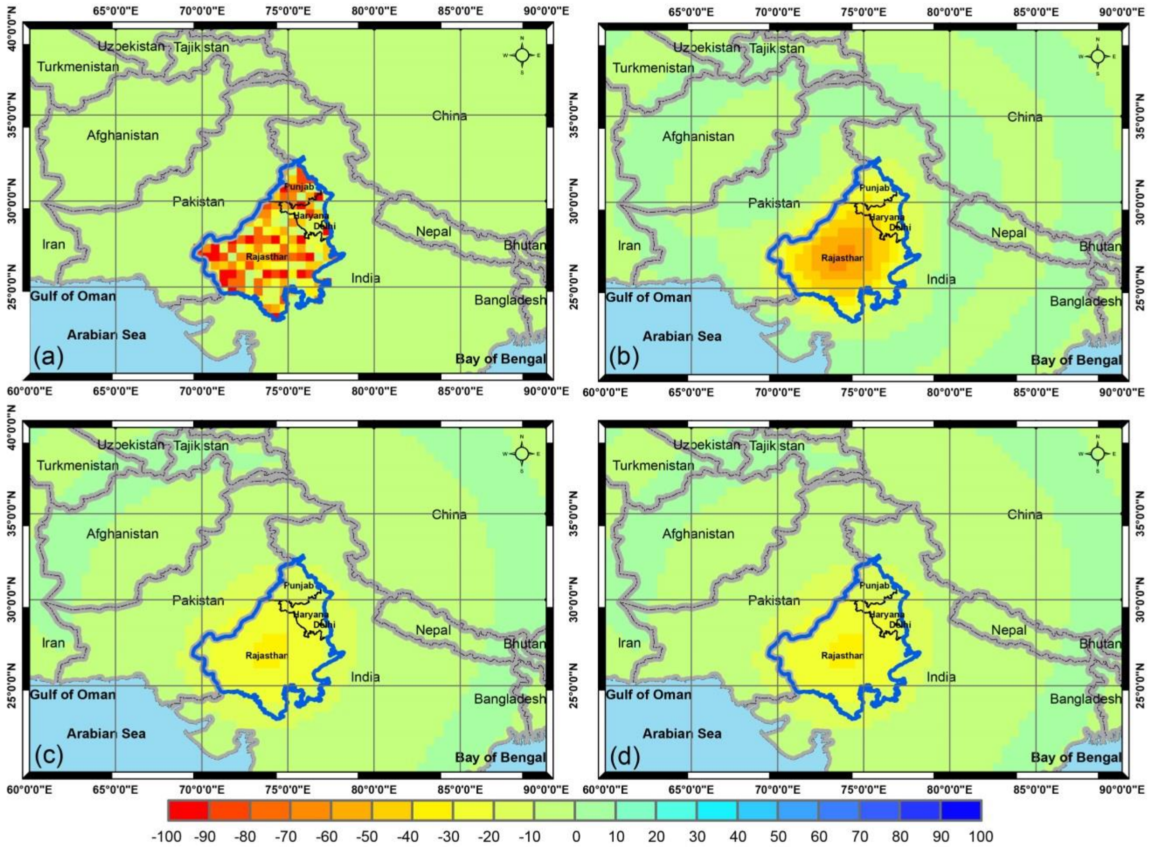

Figure S4 Evaluation of unconstrained forward modeling using (a) a hypothetic randomly distributed GWD rate with a spatial mean of -50 mm/a, (b) forward modeled GWDs rate distribution after 500 iterations, (c) GWDa derived from (a) after low-pass filtering, and (d) filtered GWDs derived from (b) after low-pass filtering. Map was created using ArcGIS (http://www.esri.com/software/arcgis/arcgis-for-desktop).

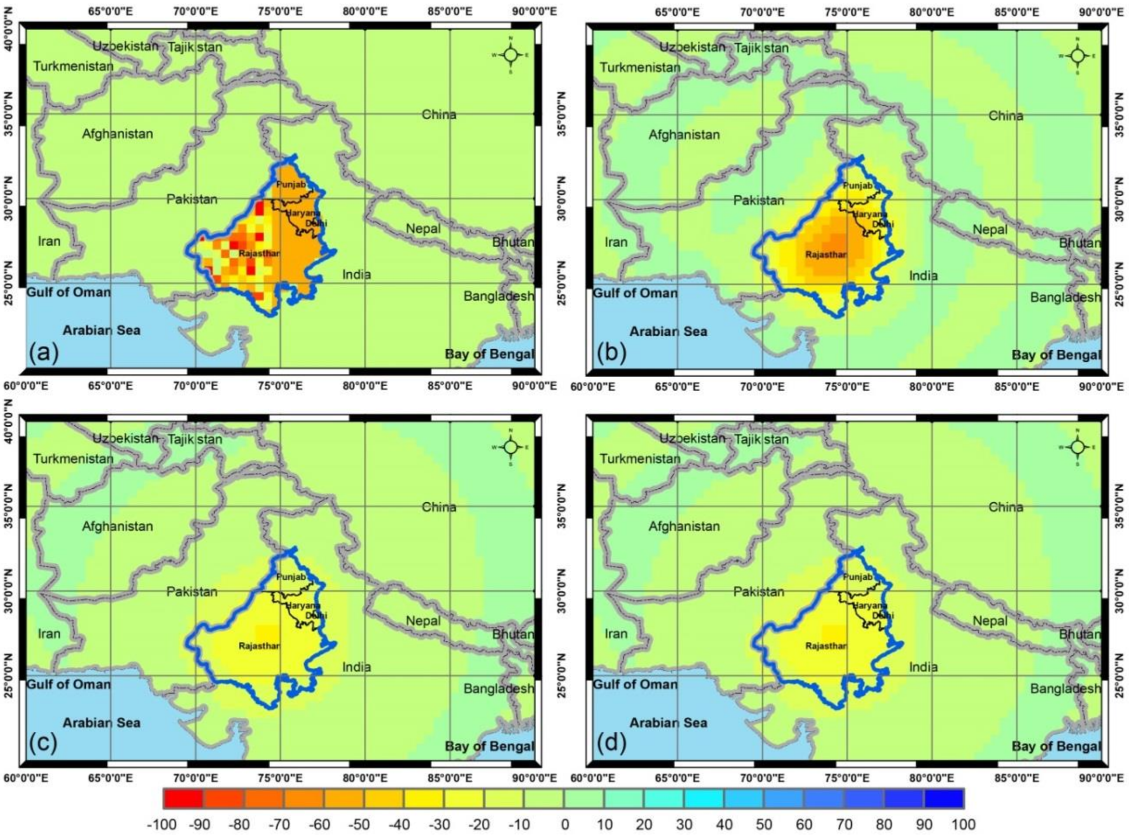

Figure S5 Evaluation of unconstrained forward modeling using (a) a hypothetic uniformly distributed GWD rate with a spatial mean of -50 mm/a for the right-half three-state region but randomly distributed GWD rates with a spatial mean of ~-50 mm/a for the other part, (b) forward modeled GWD_s rate distribution after 500 iterations, (c) GWD_a derived from (a) after low-pass filtering, and (d) filtered GWD_s derived from (b) after low-pass filtering. Map was created using ArcGIS (http://www.esri.com/software/arcgis/arcgis-for-desktop).

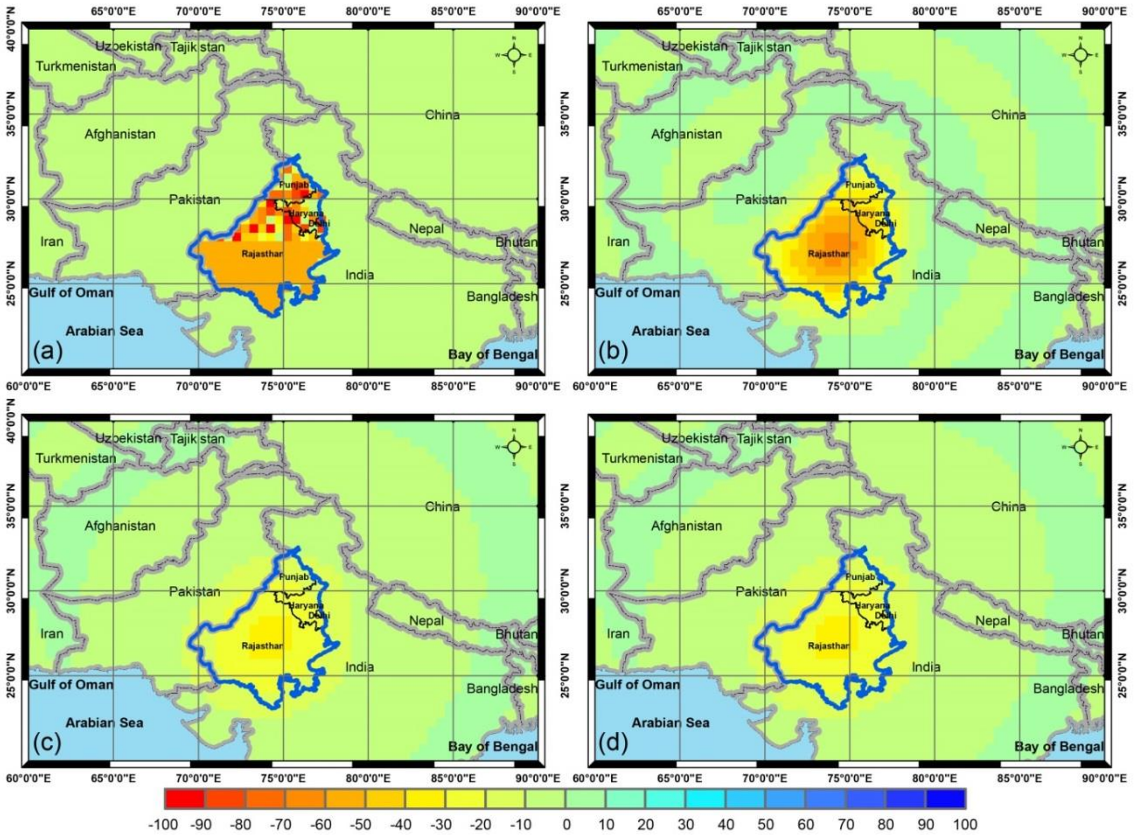

Figure S6 Evaluation of unconstrained forward modeling using (a) a hypothetic uniformly distributed GWD rate with a spatial mean of -50 mm/a for the lower-half three-state region but randomly distributed GWD rates with a spatial mean of ~-50 mm/a for the other part, (b) forward modeled GWD_s rate distribution after 500 iterations, (c) GWD_a derived from (a) after low-pass filtering, and (d) filtered GWD_s derived from (b) after low-pass filtering. Map was created using ArcGIS (http://www.esri.com/software/arcgis/arcgis-for-desktop).

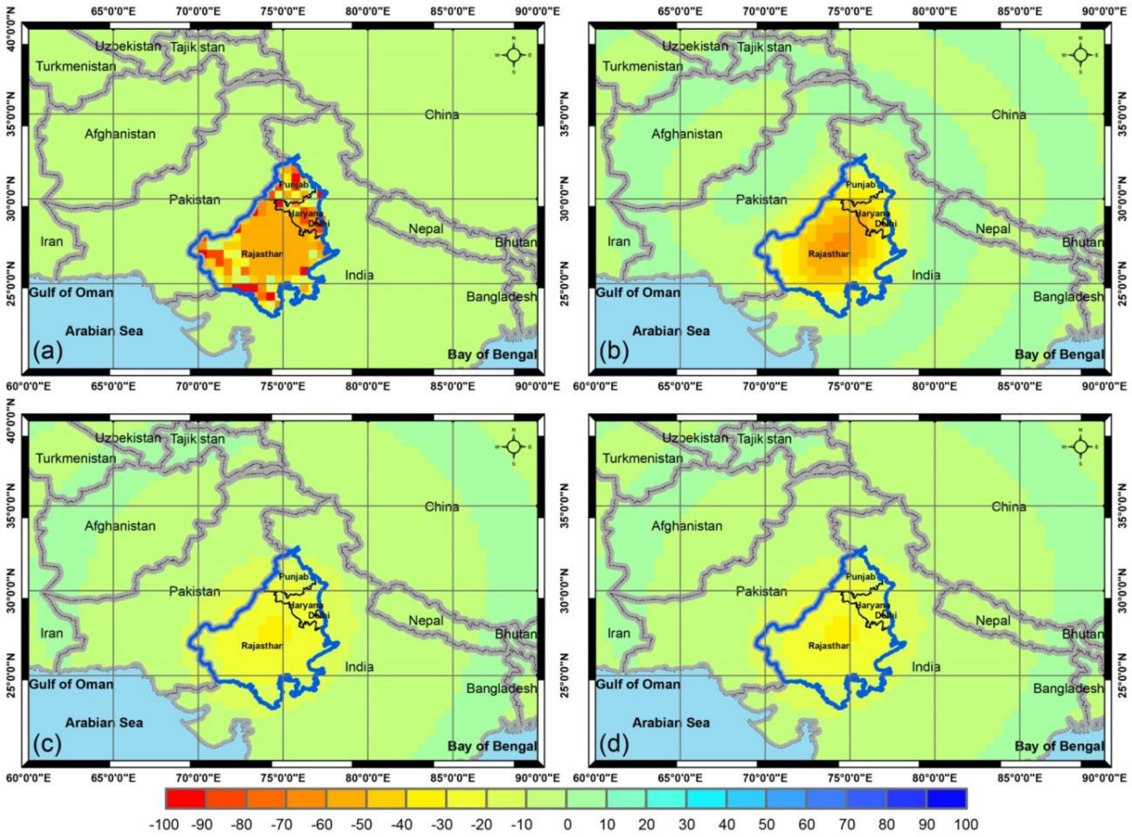

Figure S7 Evaluation of unconstrained forward modeling using (a) a hypothetic uniformly distributed GWD rate with a spatial mean of -50 mm/a for the a circular area of the three-state region but randomly distributed GWD rates with a spatial mean of ~-50 mm/a for the other part, (b) forward modeled GWD_s rate distribution after 500 iterations, (c) GWD_a derived from (a) after low-pass filtering, and (d) filtered GWD_s derived from (b) after low-pass filtering. Map was created using ArcGIS (http://www.esri.com/software/arcgis/arcgis-for-desktop).

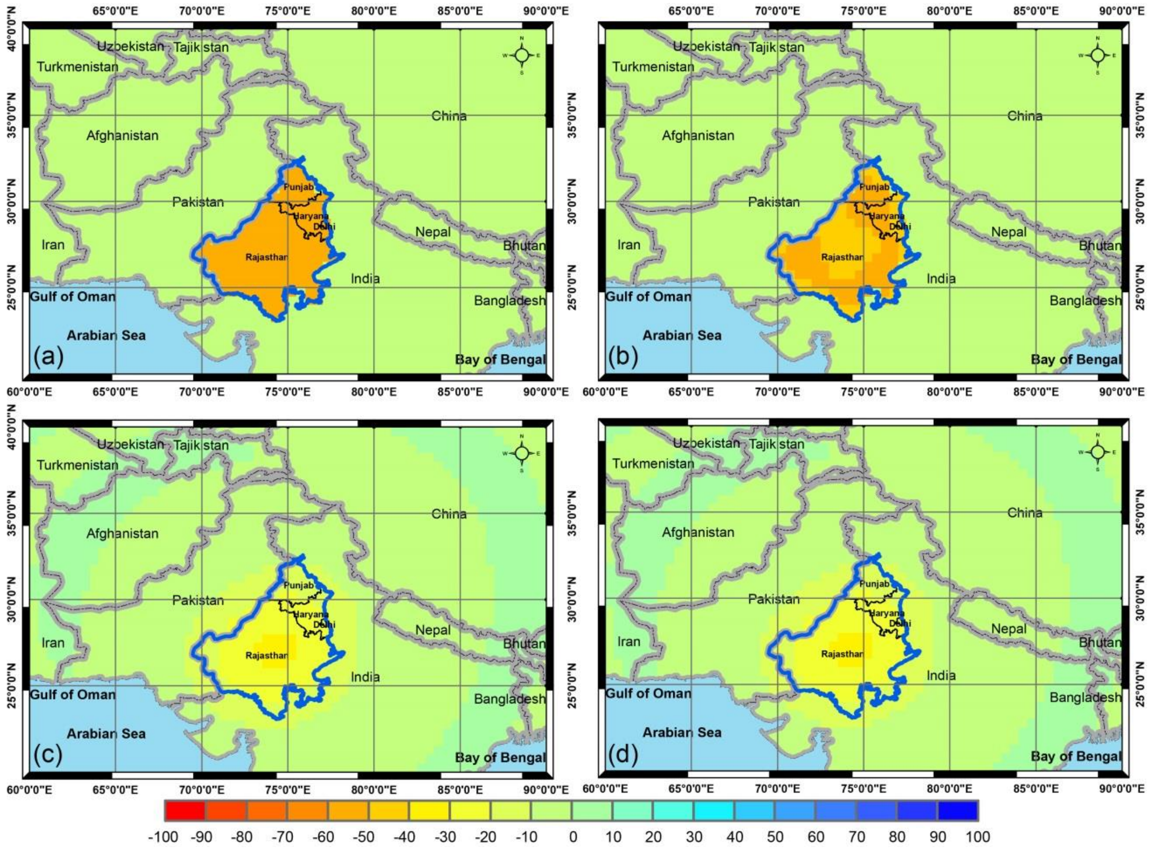

Figure S8 Evaluation of locally constrained forward modeling using (a) a hypothetic uniform distributed GWD rate with a spatial mean of -50 mm/a, (b) forward modeled GWD_s rate distribution after 500 iterations, (c) GWD_a derived from (a) after low-pass filtering, and (d) filtered GWD_s derived from (b) after low-pass filtering. Map was created using ArcGIS (http://www.esri.com/software/arcgis/arcgis-for-desktop).

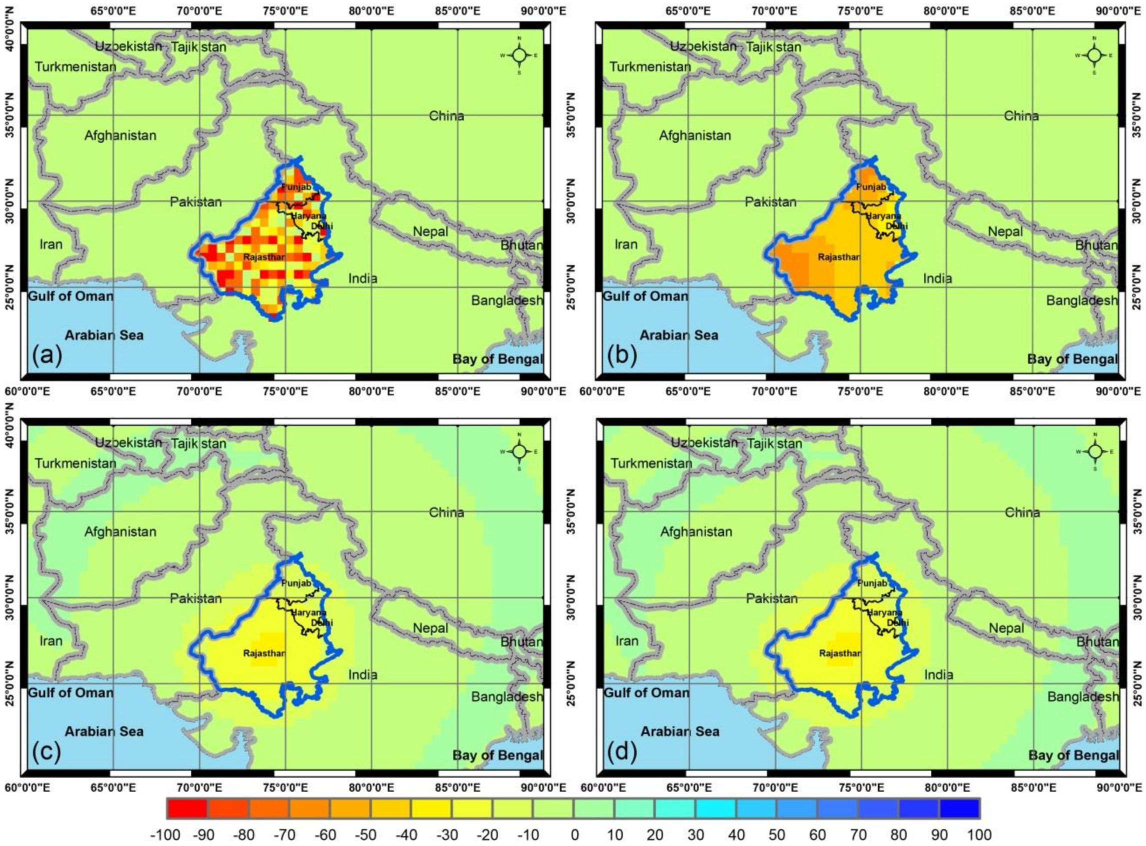

Figure S9 Evaluation of locally constrained forward modeling using (a) a hypothetic randomly distributed GWD rate with a spatial mean of -50 mm/a, (b) forward modeled GWD_s rate distribution after 500 iterations, (c) GWD_a derived from (a) after low-pass filtering, and (d) filtered GWD_s derived from (b) after low-pass filtering. Map was created using ArcGIS (http://www.esri.com/software/arcgis/arcgis-for-desktop).

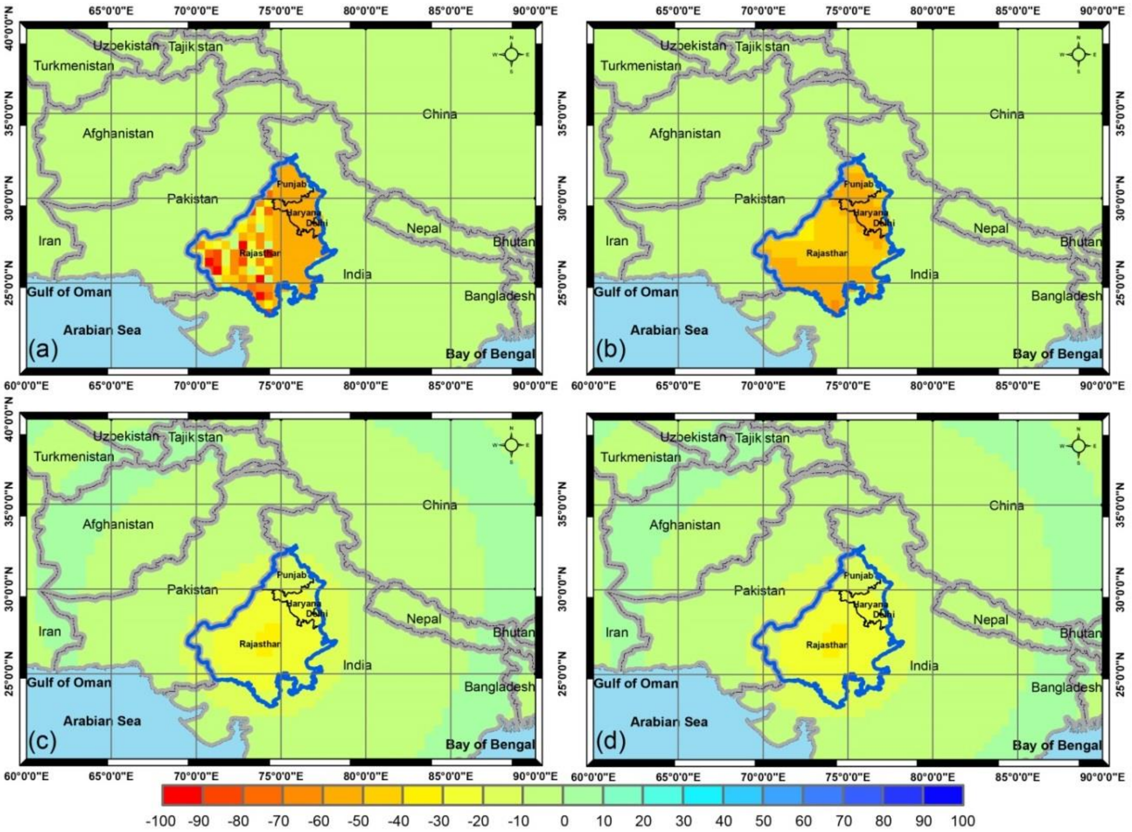

Figure S10 Evaluation of the locally constrained forward modeling using (a) a hypothetic uniformly distributed GWD rate with a spatial mean of -50 mm/a for the right-half three-state region but randomly distributed GWD rates with a spatial mean of ~-50 mm/a for the other part, (b) forward modeled GWD_s rate distribution after 500 iterations, (c) GWD_a derived from (a) after low-pass filtering, and (d) filtered GWD_s derived from (b) after low-pass filtering. Map was created using ArcGIS (http://www.esri.com/software/arcgis/arcgis-for-desktop).

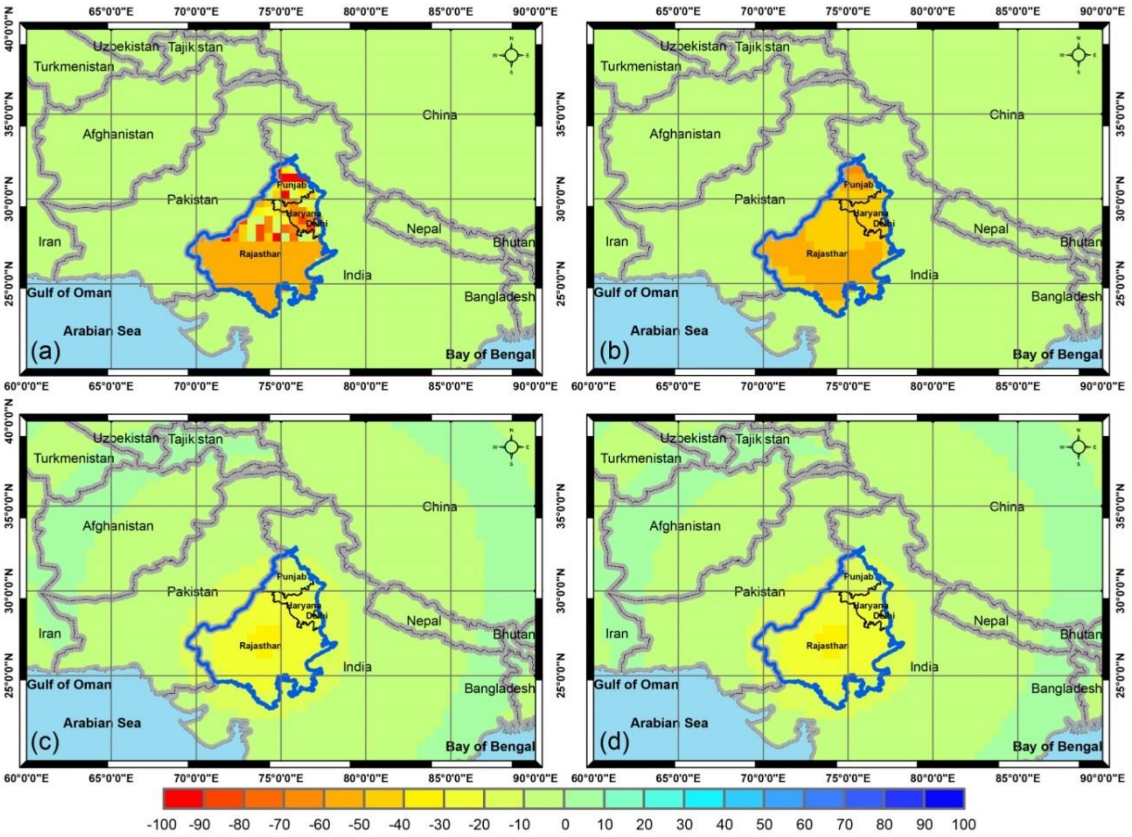

Figure S11 Evaluation of the locally constrained forward modeling using (a) a hypothetic uniformly distributed GWD rate with a spatial mean of -50 mm/a for the lower-half three-state region but randomly distributed GWD rates with a spatial mean of ~-50 mm/a for the other part, (b) forward modeled GWD_s rate distribution after 500 iterations, (c) GWD_a derived from (a) after low-pass filtering, and (d) filtered GWD_s derived from (b) after low-pass filtering. Map was created using ArcGIS (http://www.esri.com/software/arcgis/arcgis-for-desktop).

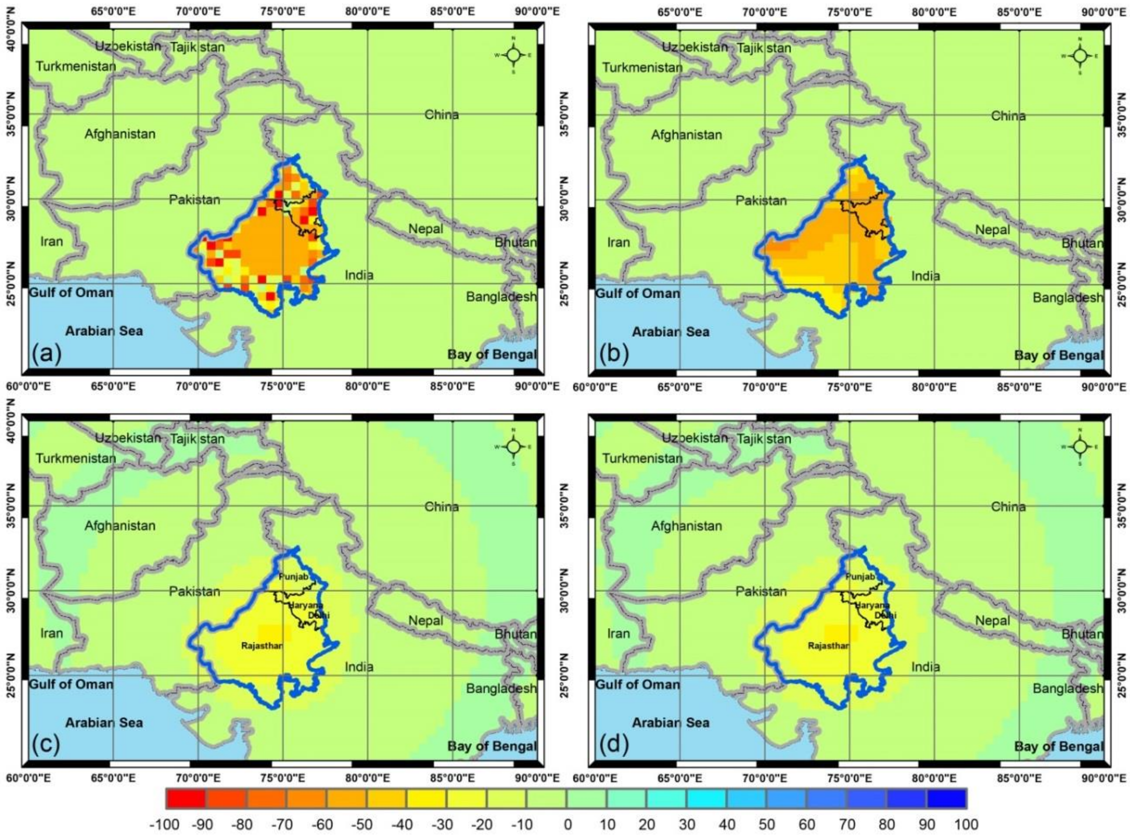

Figure S12 Evaluation of the locally constrained forward modeling using (a) a hypothetic uniformly distributed GWD rate with a spatial mean of -50 mm/a for a circular area of the three-state region but randomly distributed GWD rates with a spatial mean of ~-50 mm/a for the other part, (b) forward modeled GWD_s rate distribution after 500 iterations, (c) GWD_a derived from (a) after low-pass filtering, and (d) filtered GWD_s derived from (b) after low-pass filtering. Map was created using ArcGIS (http://www.esri.com/software/arcgis/arcgis-for-desktop).

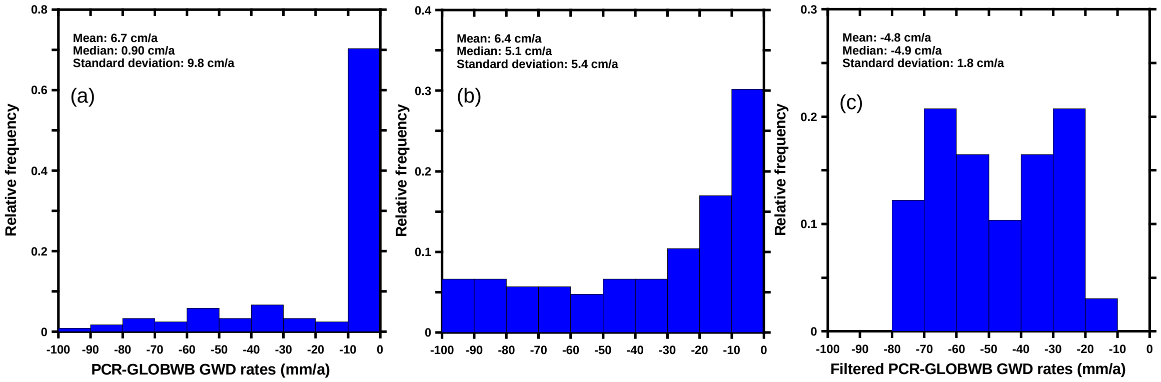

Figure S13 Relative frequency distributions of GWD rates for subplots (a), (b), and (d) in Figure 2, with showing statistics including mean, median, and standard deviation for each subplot. Map was created using SigmaPlot (http://www.sigmaplot.com/).

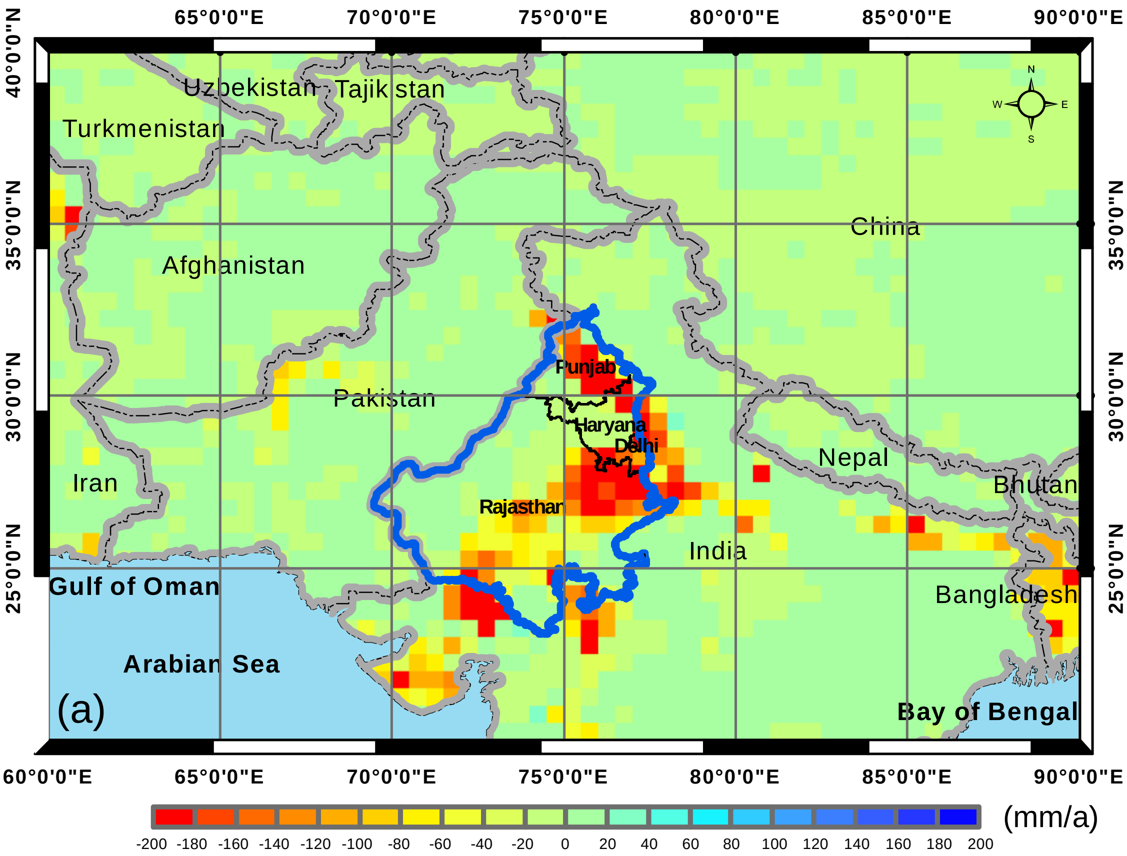

Figure S14 GWD derived from groundwater storage changes from WaterGAP Global Hydrological Model (WGHM2.2 Standard Version) for the period 2003-2009. Map was created using ArcGIS (http://www.esri.com/software/arcgis/arcgis-for-desktop).

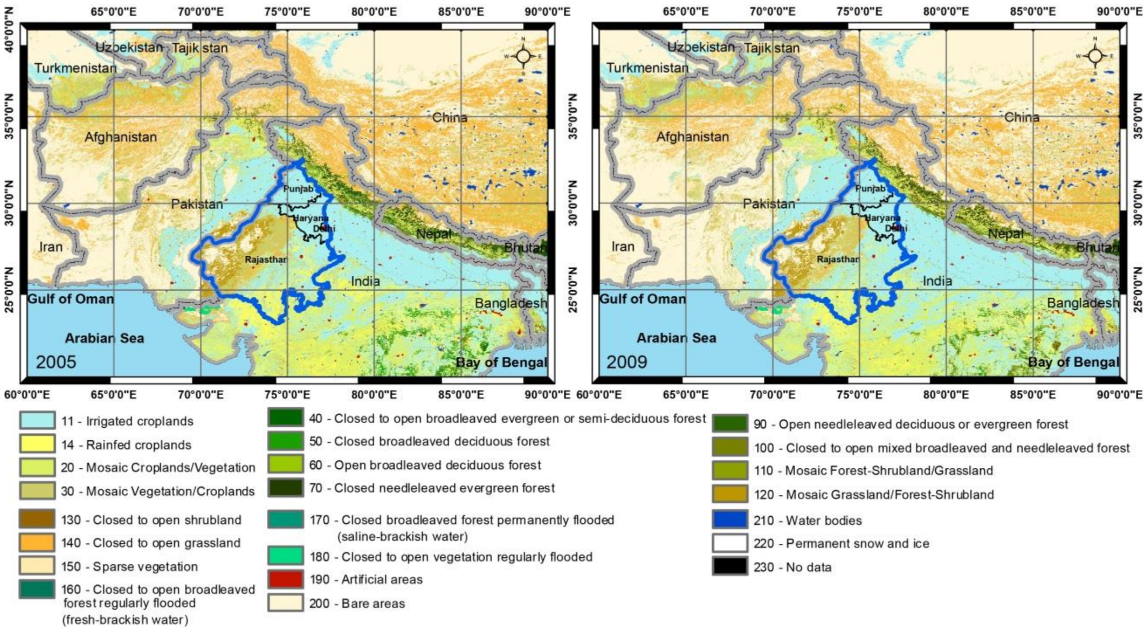

Figure S15 Land use of the three-state region for 2005 and 2009, respectively, from the European Space Agency GlobCover Portal (http://due.esrin.esa.int/globcover/). Map was created using ArcGIS (http://www.esri.com/software/arcgis/arcgis-for-desktop).

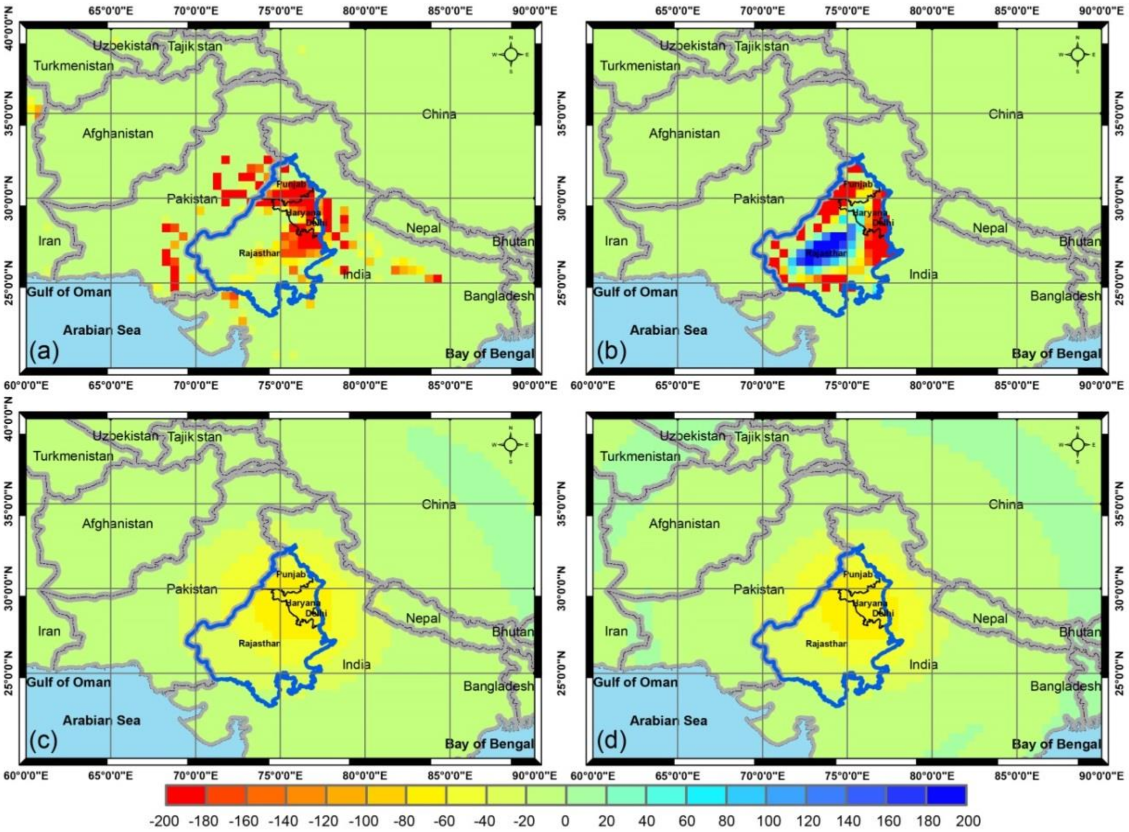

Figure S16 Evaluation of the locally constrained forward modeling using (a) synthetic GWD rates (mm/a) from PCR-GLOBWB for the period 2003-2010, (b) forward modeled GWD_s rate distribution after 500 iterations, (c) GWD_a derived from (a) after low-pass filtering, and (d) filtered GWD_s derived from (b) after low-pass filtering. Map was created using ArcGIS (http://www.esri.com/software/arcgis/arcgis-for-desktop).

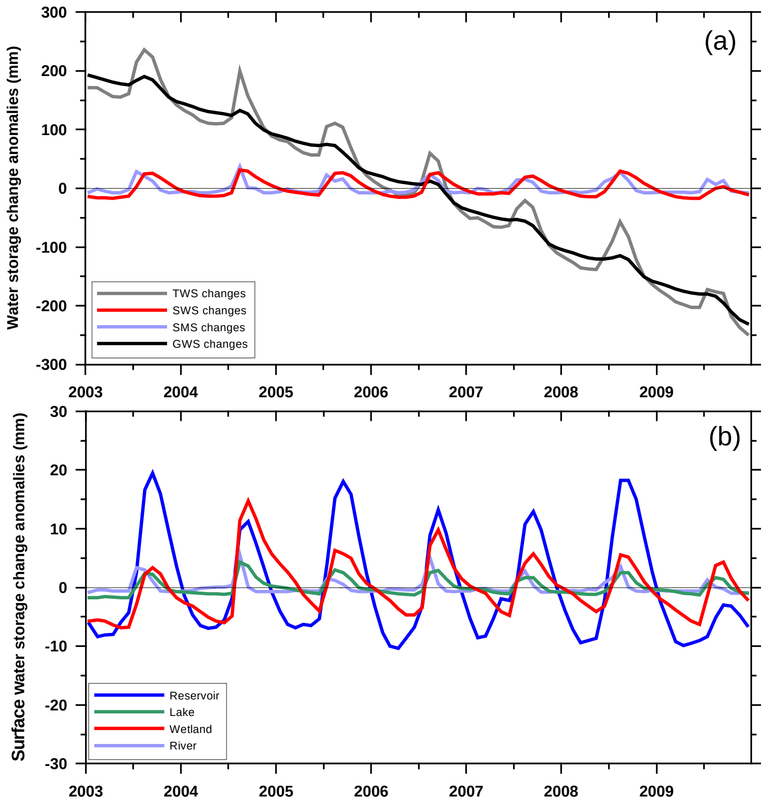

Figure S17 (a) TWS, SWS, SMS, and GWS from WaterGAP WGHM model2.2, and (b) SWS components including reservoir, lake, wetland, and river storage changes from WGHM. Map was created using SigmaPlot (http://www.sigmaplot.com/).

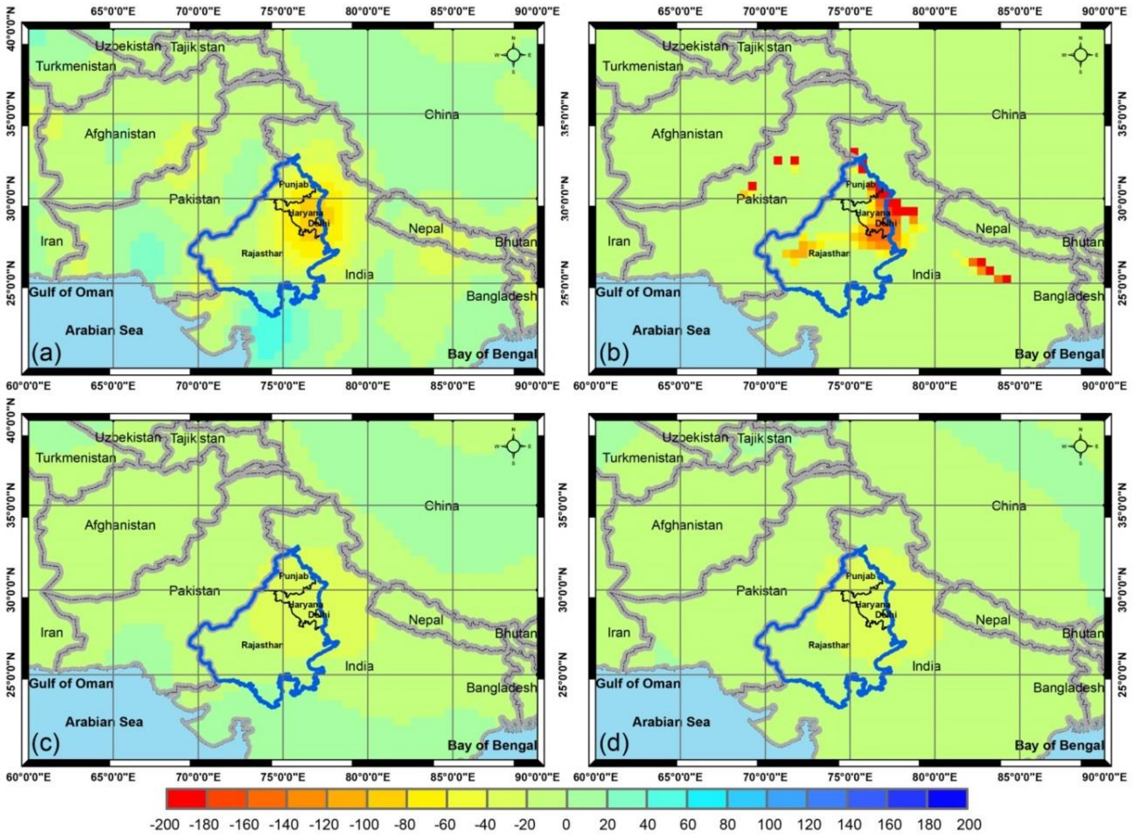

Figure S18 (a) GWD rates from original GRACE TWS changes without filtering minus GLDAS SMS changes for the period 2003-2010; (b) GWD_s rates from regionally constrained forward modeling after 500 iterations using the spatial pattern of GWD rates from PCR-GLOBWB for the same period, (c) filtered GRACE GWD rates, i.e., GWD_a, and (d) filtered GWD_s derived from (b) after low-pass filtering. Map was created using ArcGIS (http://www.esri.com/software/arcgis/arcgis-for-desktop).

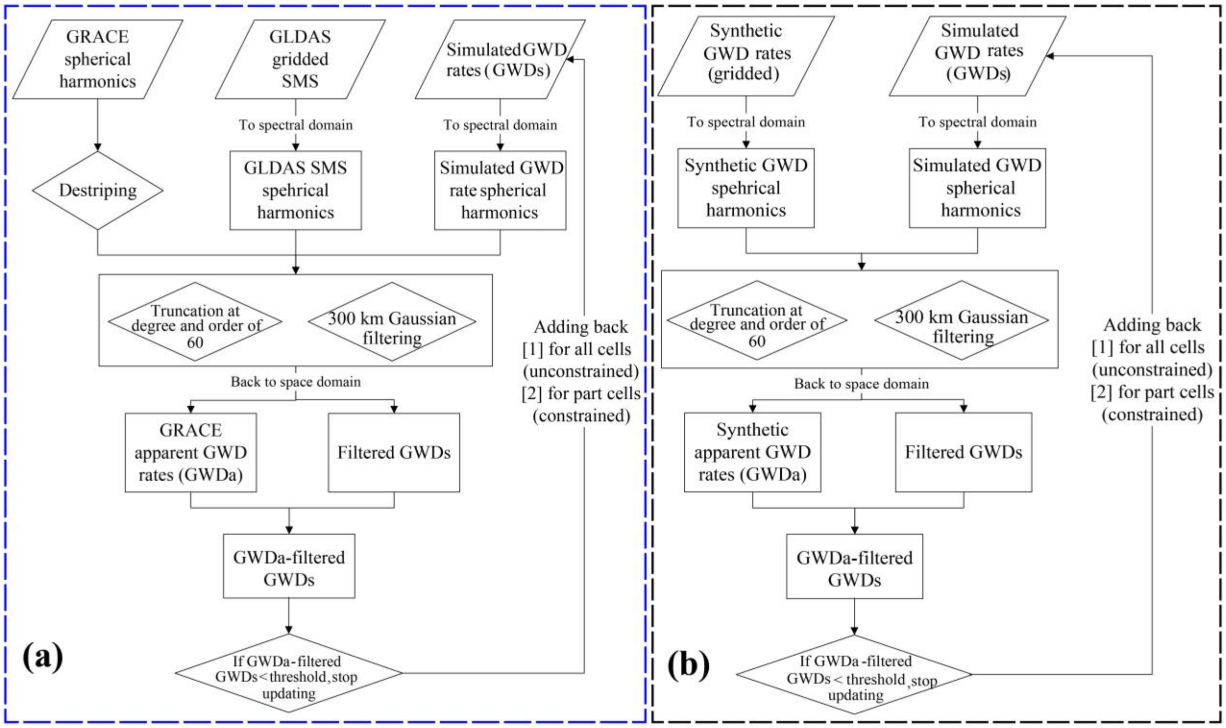

Figure S19 A schematic showing (a) GWD estimation from GRACE satellites and (b) signal restoration for filtered GWD from synthetic data as a case using unconstrained and constrained forward modeling. Map was created using MS Office Visio (https://products.office.com/en-us/visio/flowchart-software).

Table S1 Statistics of original, recovered and filtered signals (mm/a) from both unconstrained and (globally and locally) constrained forward modeling after 500 iterations for five configurations of hypothetical data for the three-state region of India

Hypothetical configuration of original data		Uniform (1)	Random (2)	Right-half (3)	Lower-half (4)	Circle (5)	PCR
Hypothetical/ synthetic data	Spatial mean	-50	-50.4	-48.2	-51.0	-49.1	-67.4
Unconstrained	Recovered spatial mean	-39.0	-38.6	-37.8	-39.8	-38.3	-64.3
	Filtered original data	-23.4	-23.3	-22.7	-23.9	-23.0	-47.5
	Correlation between recovered and original signals	0.22	0.04	0.13	0.13	0.12	0.64
Hypothetical/ synthetic data	Spatial mean	-50	-50.4	-49.5	-49.6	-48.6	-67.4
Globally (locally) constrained	Recovered spatial mean	-50	-50.3	-49.5	-49.6	-48.6	-68.4 (-121.8)
	Filtered original data	-23.4	-23.3	-23.0	-23.1	-22.8	-47.5 (-47.4)
	Correlation between recovered and original signals	0.99	0.36	0.48	0.48	0.45	0.77 (0.28)

Table S2 Comparison of basic model configuration of LSMs and GHMs and its outputs used in this study

Model	SWS	SMS<br>No. of layer and total depth (m)	GWS	Spatial resolution (°, km)	Temporal resolution	Timespan	Human impacts on water storage
GLDAS-1 Noah 2.7	Snow and canopy	4, 2	No	0.25, ~28	Three hours; monthly	2003-2012	No
GLDAS-1 Mosaic		3, 3.5	No	1, ~111			
GLDAS-1 VIC		3, 1.9	No	1, ~111			
GLDAS-1 CLM2.0		10, 3.4	No	1, ~111			
PCR-GLOBWB	Snow, canopy, reservoirs, rivers, lakes, wetlands, canopy	2, 1.5	Yes	0.5, ~55	Daily; monthly	2003-2010	- Water use for livestock, irrigation, industrial, and domestic sectors<br>- Surface water and groundwater abstraction<br>- Irrigation return flow as part of groundwater recharge<br>- Reservoir regulation
WGHM 2.2	Snow, canopy, reservoirs, rivers, lakes, wetlands, canopy	1, depending on land cover and according rooting depth	Yes	0.5, ~55	Daily; monthly	2003-2009	- Water use for livestock, irrigation, manufacturing, cooling for thermal power plants, and domestic sectors<br>- Surface water and groundwater abstraction<br>- Irrigation return flow as part of groundwater recharge<br>- Reservoir regulation

References

1 Longuevergne, L., Scanlon, B. R. & Wilson, C. R. GRACE Hydrological estimates for small basins: Evaluating processing approaches on the High Plains Aquifer, USA. *Water Resour*. *Res*. **46**; DOI: 10.1029/2009wr008564 (2010).

2 Landerer, F. W. & Swenson, S. C. Accuracy of scaled GRACE terrestrial water storage estimates. *Water Resour Res* **48**; DOI:10.1029/2011wr011453 (2012).

3 Klees, R., Zapreeva, E. A., Winsemius, H. C. & Savenije, H. H. G. The bias in GRACE estimates of continental water storage variations. *Hydrology and Earth System Sciences* **11**, 1227-1241 (2007).

4 Döll, P., Müller Schmied, H., Schuh, C., Portmann, F. T. & Eicker, A. Global-scale assessment of groundwater depletion and related groundwater abstractions: Combining hydrological modeling with information from well observations and GRACE satellites. *Water Resour*. *Res*. **50**, 5698-5720 (2014).

5 Fenoglio-Marc, L., Kusche, J. & Becker, M. Mass variation in the Mediterranean Sea from GRACE and its validation by altimetry, steric and hydrologic fields. *Geophysical Research Letters* **33**; DOI: 10.1029/2006gl026851 (2006).

6 Velicogna, I. & Wahr, J. Measurements of time-variable gravity show mass loss in Antarctica. *Science* **311**, 1754-1756 (2006).

7 Swenson, S. & Wahr, J. Multi-sensor analysis of water storage variations of the Caspian Sea. *Geophysical Research Letters* **34**; DOI: 10.1029/2007gl030733 (2007).

8 Chen, J. L., Wilson, C. R., Li, J. & Zhang, Z. Reducing leakage error in GRACE-observed long-term ice mass change: a case study in West Antarctica. *J. Geodesy*, 1-16; DOI:10.1007/s00190-015-0824-2 (2015).

9 Chen, J. L., Li, J., Zhang, Z. Z. & Ni, S. N. Long-term groundwater variations in Northwest India from satellite gravity measurements. *Global Planet Change* **116**, 130-138 (2014).

10 Rodell, M., Velicogna, I. & Famiglietti, J. S. Satellite-based estimates of groundwater depletion in India. *Nature* **460**, 999-1002 (2009).

11 Velicogna, I. & Wahr, J. Acceleration of Greenland ice mass loss in spring 2004. *Nature* **443**, 329-331 (2006).

12 Swenson, S. & Wahr, J. Post-processing removal of correlated errors in GRACE data. *Geophysical Research Letters* **33**; DOI: 10.1029/2005gl025285 (2006).

13 Chen, J. L., Wilson, C. R., Famiglietti, J. S. & Rodell, M. Spatial sensitivity of the Gravity Recovery and Climate Experiment (GRACE) time-variable gravity observations. *J. Geophys*. *Res-Sol Ea* **110**; DOI: 10.1029/2004jb003536 (2005).

14 Chen, J. L., Wilson, C. R., Blankenship, D. & Tapley, B. D. Accelerated Antarctic ice loss from satellite gravity measurements. *Nature Geosci*. **2**, 859-862 (2009).

15 Long, D., Longuevergne, L. & Scanlon, B. R. Global analysis of approaches for deriving total water storage changes from GRACE satellites. *Water Resour*. *Res*. **51**, 2574-2594 (2015).

16 Siebert, S. *et al.* Groundwater use for irrigation – a global inventory. *Hydrol. Earth Syst. Sci.* **14**, 1863-1880 (2010).

17 Famiglietti, J. S. *et al.* Satellites measure recent rates of groundwater depletion in California's Central Valley. *Geophys. Res. Lett.* **38**, L03403; DOI:10.1029/2010gl046442 (2011).

18 Müller Schmied, H. *et al.* Sensitivity of simulated global-scale freshwater fluxes and storages to input data, hydrological model structure, human water use and calibration. *Hydrol. Earth Syst. Sci.* **18**, 3511-3538 (2014).

19 Ministry of Water Resources, Government of India. Report of the ground water resource estimation committee: Ground water resource estimation methodology <http://cgwb.gov.in/documents/gec97.pdf> (2009) Date of access: 31/08/2015.

20 Dhiman, S.C. Aquifer systems of India. Ministry of Water Resources, Government of India. <http://www.cgwb.gov.in/AQM/India.pdf> (2012) Date of access: 31/08/2015.

21 Alcamo, J. *et al.* Development and testing of the WaterGAP 2 global model of water use and availability. *Hydrological Sciences Journal-Journal Des Sciences Hydrologiques* **48**, 317-337 (2003).

22 Doll, P., Kaspar, F. & Lehner, B. A global hydrological model for deriving water availability indicators: model tuning and validation. *J. Hydrol*. **270**, 105-134 (2003).

23 Doll, P. & Siebert, S. Global modeling of irrigation water requirements. *Water Resour*. *Res*. **38**; DOI: 10.1029/2001wr000355 (2002).

24 Vassolo, S. & Doll, P. Global-scale gridded estimates of thermoelectric power and manufacturing water use. *Water Resour*. *Res*. **41**; DOI: 10.1029/2004wr003360 (2005).

25 Lehner, B. & Doll, P. Development and validation of a global database of lakes, reservoirs and wetlands. *J. Hydrol*. **296**, 1-22 (2004).

26 Doll, P. *et al.* Impact of water withdrawals from groundwater and surface water on continental water storage variations. *J. Geodyn*. **59**, 143-156 (2012).
